# Geographic Targeting and Normative Frames: Revisiting the Equity of Conditional Cash Transfer Program Distribution in Bolivia, Colombia, Ecuador, and Peru

**DOI:** 10.1186/s12939-020-01233-0

**Published:** 2020-07-31

**Authors:** Mathieu J. P. Poirier

**Affiliations:** 1 School of Global Health, Faculty of Health, York University, 4700 Keele Street, Dahdaleh Building 5022C, Toronto, Ontario M3J 1P3 Canada; 2 Global Strategy Lab, York University, 4700 Keele Street, Dahdaleh Building 5022C, Toronto, Ontario M3J 1P3 Canada

**Keywords:** Program Targeting, Conditional Cash Transfer, Normative Frames, Health Equity, Socioeconomic Status, Bolivia, Colombia, Ecuador, Peru

## Abstract

**Background:**

Four Andean countries of Bolivia, Colombia, Ecuador, and Peru introduced national health-focused conditional cash transfer (CCT) programs in the 2000s. This study probes whether policymakers in these countries targeted CCT programs to subregions with the highest prevalence of ill-health or those with the lowest socioeconomic status (SES) to evaluate the equity of geographic targeting and means-testing, as well as the potential role of normative frames, bounded rationality, and clientelism as explanatory mechanisms for inequities in social spending.

**Methods:**

The distribution of vaccination coverage, underweight, stunting, and child deaths is established both within and between subnational regions and SES quintiles from 1998 to 2012 using every available nationally representative household survey. The equity of CCT program targeting and strength of association with subregional SES and health outcomes are measured using generalized entropy index decomposition and meta-regression. Finally, simple predictive models for CCT targeting are created using lagged subregional SES, health outcomes, and concentration indices.

**Results:**

Bolivia and Peru both effectively targeted at-risk subregions, but subregions in Peru with no CCT program coverage result in higher mistargeting rates for the country as a whole. Only Bolivia failed to attain CCT coverage concentration indices that are at least as large as the health inequalities they are targeting. Despite this insufficient progressivity, Bolivia has the most efficient subregional targeting, while the lowest rates of mistargeting for child deaths are found in Colombia and Ecuador. Finally, the simple predictive model performs as well or better than observed CCT coverage distribution for every country, year, and outcome.

**Conclusions:**

Both Peru and Ecuador have targeted programs to their poorest populations effectively, demonstrating that this is possible with both universal and geographic targeting. No clear evidence of clientelism was found, while the dominant normative frame underlying CCT program targeting decisions appears to be the relative SES of subregions, rather than absolute SES, prevalence of health outcomes, or health inequalities. To reduce the inequitable impacts of bounded rationality, policymakers can use simple predictive models to target CCT coverage effectively and without leaving behind the most vulnerable populations that happen to live in more affluent subregions.

## Background

Policymakers face difficult choices when designing and implementing policies meant to address health inequalities. The way policymakers perceive determinants of health and cope with information gaps at the time of decision-making can have a large effect on the impacts these policies will have. While these inner thought processes (i.e. normative frames), [1–3] the consequences of making decisions with less than perfect information (i.e. bounded rationality), [1, 4] and hidden motivations (e.g. clientelism) [5, 6] can never be known with certainty, it is possible to probe the potential impacts of these effects with careful study of illustrative cases. Operating under the assumption that distinct policy choices made under similar conditions provide insights into the real-world consequences of these cognitive frames, this study probes four national health-focused conditional cash transfer (CCT) programs to investigate the degree to which each country’s program targeting differed from both hypothetically perfect and evidence-informed counterfactuals.

Among policies meant to improve health equity, CCTs are unique in attempting to address both socioeconomic and health dimensions at the same time. Progressivity is often built into the targeting of CCT programs through income- or consumption-based means-testing, geographic targeting, age or gender targeting, or by combining these factors into an aggregate index [7]. Once the target population is identified, conditionalities, which could include minimum school attendance, giving birth at a health center, bringing infants to health checkups, receiving vaccinations, or attending community social outreach programs must be met as a prerequisite for receiving a cash transfer [8, 9]. The size of cash benefits varies between countries, but are generally intended to offset opportunity costs associated with the conditional behavior [10]. Although the long-term goals of these programs, and thus, the effectiveness of reaching those goals is disputed, [11] there is evidence that these programs are achieving progress in improving health, school attendance, and social cohesion while reducing poverty with low administrative costs and rapid implementation [7, 12–18].

Decisions about how best to target CCT programs are often driven by normative framing of health and socioeconomic status (SES). In part, this is because quantifying SES is more complicated than simply asking for annual income in many low- and middle-income countries. Factors such as informal employment and large fluctuations in income from month to month have led many researchers, and in turn policymakers, to prefer household consumption or wealth indices as more reliable measures of SES [19–21]. Moreover, policymakers must weigh the relative importance of both absolute and relative inequalities measured by each of these SES measures. Similarly, population health can be measured in many ways, but can broadly be broken down into categories of process indicators like vaccination rates and births occurring in health facilities, and direct health outcomes ranging from acute to long-term conditions. Decisions made by policymakers can be affected by whether their subconscious framing of health inequality is an issue of access (poor households cannot access vaccinations for their children), or an issue of outcomes (poor children are more likely to die before the age of five).

Targeting the health promotion and cash benefits to those that would most benefit from them can be even more difficult in contexts with limited resources and information. While the link between adverse health outcomes and low SES has been demonstrated in nearly every setting in the world, the strength of association between the two can vary greatly both within and between countries [22–25]. In very low-income settings, the magnitude of health inequalities is often smaller than higher income settings, which is driven by broadly distributed ill health among the large proportion of the population that is poor in absolute terms [26, 27]. That means that even when some population-level information is known, targeting populations with ill health living in poverty with national CCT programs must balance the absolute distribution of both ill health and poverty, and cannot always rely on subnational health inequality indicators.

In an ideal world, CCT programs would be resourced to allow for full implementation in every part of the country and for every recipient to be individually assessed for need. However, when resources are limited, programs are often targeted geographically or the implementation of means-testing not fully resourced [28]. In theory, geographic targeting of programs can be efficient if there is wide variation in health and wealth between subnational regions, however, poor households in richer regions will inevitably be left behind. Passive means-testing can be conducted by asking potential beneficiaries to self-identify and prove their eligibility, but ineffective program outreach can result in the households with the most need not receiving assistance they are entitled to [29]. Finally, it is always possible that this mistargeting is not due to chance, but driven by clientelist, electoral, financial, social, or economic factors.

This study leverages administrative and household survey data evaluating CCT programs created between 2003 and 2009 in four contiguous Andean countries to investigate the potential effects of policymakers’ cognitive frames in targeting CCT programs. Health inequalities at the subnational level are measured using administrative program data and every nationally representative household health survey available since 1998 to investigate how the targeting of these programs varied between countries. Lastly, this study compares actual targeting of CCT programs in each country against both a hypothetical perfectly targeted model and a simple data-driven model using information available to policymakers at the time of annual program implementation. Together, these analyses probe how policymakers in these four countries frame the issue of inequalities in health and cope with missing information and limited resources.

## Methods

The objective of the case selection strategy was to identify contemporaneous national CCT programs in a defined region to provide more confidence that differences in the distribution of health and SES, and not temporal or cultural factors, were driving decision making, while also including a diverse set of approaches to geographic targeting and means-testing. This diverse-case purposive sampling strategy led to the wave of Latin American CCT programs that were rolled out in the 2000s in the wake of two highly touted programs of *Bolsa Familia* in Brazil and *Progresa* in Mexico [30–32]. Within this region, four contiguous Andean countries of Colombia, Ecuador, Peru, and Bolivia were identified that had all developed health-focused CCTs within a period of 6 years, but were evenly split between strong and weak means-targeting, and geographic targeting and universal rollout. Other Andean countries were considered for inclusion, but Venezuela was lacking in reliable longitudinal data, while Argentina and Chile’s relatively high SES and more complex welfare state architecture would have complicated regional comparisons [8, 33, 34]. A focus on Central American countries was also considered, but the smaller scale of these countries’ CCT programs, as well as the early discontinuation or lack of explicit focus on health of some programs would have resulted in a less informative approach than a focus on the Andean region [8, 35, 36].

The four CCT programs under study (Table 1) are united in their focus on improving maternal and child health using cash transfers conditioned on specific health-promoting co-responsibilities. Colombia’s *Más Familias en Acción* (MFA) is a multifaceted CCT program intended to improve health and educational outcomes, targeting poor households and internally displaced victims of violence [18, 38, 39]. Ecuador revamped and expanded the *Bono Solidario* into the *Bono de Desarrollo Humano* (BDH) in 2003, incorporating CCTs to improve health and educational outcomes among poor families [14, 42]. Peru’s *Juntos* was created in 2005, seeking to break the intergenerational transmission of poverty by improving the health and education of children [16, 43]. Finally, Bolivia created the *Bono Juana Azurduy* (BJA) in 2009 to improve maternal and child health [12]. Taken together, two of the CCT programs relied on geographic targeting (MFA and *Juntos*) and three programs developed a quantitative instrument to target vulnerable households (MFA, BDH, and *Juntos*). Moderate differences in health and wealth between these four countries detailed in Table 1 are to be expected with a purposive diverse-case sampling strategy and allow for the generation of information on whether policymakers use geographic targeting and means-testing to maximize scarce resources, and whether broadly distributed ill health and lower information quality make these strategies a rational choice.

**Table 1:** Key country and CCT program characteristics for Bolivia, Colombia, Ecuador, and Peru

Country	GDP per capita, PPP [33]	Infant Mortality Rate [33]	CCT Program (year launched)^**a**^	CCT Focus	Targeting mechanism	Target population	Program enrollment (budget in $US)
Bolivia	$7081	29.2	*Bono Juana Azurduy* (2009)	Health	Universal, weak means-testing	All pregnant women who report not having social protection coverage [37]	127 thousand beneficiaries ($33 million)^b^ [12]
Colombia	$12,982	14.9	*Más Familias en Acción* (2006)	Health and education	Geographic, weak means-testing	SISBEN household vulnerability index based on health, education, household, and vulnerability indicators and residence in specified municipal groups [18, 38, 39]	2.7 million families ($1149 million)^c^ [38]
Ecuador	$11,431	14.4	*Bono de Desarrollo Humano* (2003)	Health and education	Universal, strong means-testing	Selben index based on infrastructure, demographics, education, and household assets [13]	1.2 million beneficiaries ($481 million)^d^ [40]
Peru	$11,176	14.3	*Juntos (2005)*	Health and education	Geographic, strong means-testing	Several iterations of district and household level indices based on health, education, demographic, and wealth indicators [17, 41]	650 thousand households ($324 million)^e^ [17, 41]

a Although Colombia’s MFA was originally created in 2000, it was massively expanded and revamped in 2006. Ecuador’s BDH represents a significant expansion in budget and scope of the *Bono Solidario*, originally created in 1998.

b Bolivia data are for 2013

c Colombia data are for 2014

d Ecuador data are for 2012

e Peru data are for 2012

**Fig. 1:**
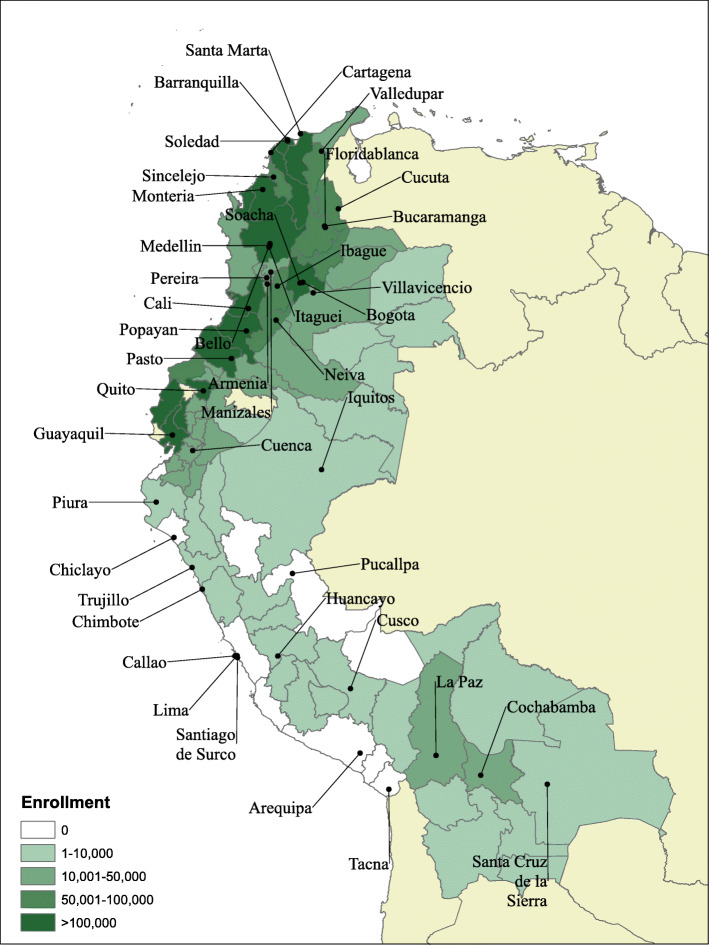
Number of adult CCT enrollees per subregion for Bolivia, Colombia, Ecuador, and Peru

There are a total of 88 subnational regions throughout the four countries, with 9 Departments in Bolivia, 33 Departments in Colombia, 21 Provinces in Ecuador, and 25 Provinces in Peru. Although the populations of each subregion range from 30,000 to 9,000,000, each country has a similar range of population distributions with at least one smaller subregion of approximately 100,000 inhabitants and the largest subregion containing more than 2,500,000 people. In order to measure outcome data, every subregionally-representative health survey since 1998 was included for analysis. For Bolivia this included Demographic and Health Surveys (DHS) for 1998, 2003, and 2008, a Multiple Indicator Cluster Survey from 2000, and the *Encuesta de Evaluación de Salud y Nutrición* (ESNUT) from 2012. For Colombia, three DHS survey rounds were used from 2005, 2010, and 2015.^1^ The *Encuesta demográfica y de salud materna e infantil* (ENDEMAIN) from 1999 and 2004 and the 2012 Encuesta Nacional de Salud y Nutrición (ENSANUT) were used for Ecuador. Finally, six rounds of DHS from 2000, 2004, 2009, 2010, 2011, and 2012 were available for Peru. Enrollment data at the subnational region level for each CCT program (Fig. 1) was obtained from the government agencies responsible for implementation in each country. Survey-reported CCT program enrollment was used to calculate household-level inequalities, while administrative data was used for all regression-based analyses.

Health outcomes were selected to be comparable between countries and to be relevant to the four CCT programs, which broadly cover maternal and child health. Prevalence of third dose of diphtheria, pertussis, and tetanus (DPT) or pentavalent vaccine was selected as a process indicator of public health protection for children.^2^ Direct health outcomes ranged from shorter term to longer term, with underweight used as an indicator of acute malnutrition, stunting as an indicator of chronic malnutrition, and ratio of child deaths to births reported by women aged 15–49 as an indicator of mortality.^3^ Age-standardized weight-for-age and height-for-age z-scores using DHS standards based on WHO Anthro definitions were used to calculate prevalence of underweight and stunting [44, 45]. Finally, household wealth indices were calculated for non-DHS household surveys using the standard DHS principal components analysis method [46–48].

The first analyses were conducted to break down inequalities in health outcomes and CCT targeting according to the proportions explained by household SES and geography. In order to do this, the proportion of inequalities in vaccine coverage, underweight, stunting, and child deaths attributable to residence in a geographic subregion and to household SES quintile was decomposed using a generalized entropy index [49, 50]. Generalized entropy indices are information theoretic measures of inequality that have the useful property of additive decomposability, meaning that the sum of within-group and between-group inequality is equal to total inequality [51]. Therefore, it is not the generalized index value itself that is of interest (because it is purely a function of prevalence), but the percentage that is additively decomposed to within- and between-subregion and to within- and between-SES quintile components [51, 52]. As a separate measure of SES-related health and CCT coverage inequalities, concentration indices (CIs) were calculated for each outcome [53, 54]. CIs are bivariate measures of inequality which can be conceptualized as being equal to the value of two times the area between a concentration curve and the line of equality; or the proportion of the outcome that would have to be redistributed from the richest half to the poorest half of the population [54, 55]. Key to this analysis is that in order for a CCT program to be sufficiently progressive to reach all of those in need, the CCT coverage CI must be at least as large as the health outcome CI. In other words, a larger CI for the health outcome than the CCT coverage would mean that even if the program was perfectly targeted to only households in relative poverty that had the health outcome of interest, there would still be poor households with the health outcome that lacked coverage.

In order to investigate plausible cognitive frames underlying policymaker’s decision making in targeting CCT coverage, one-year lagged subregion SES, one-year lagged subregion health outcomes, and one-year lagged subregion health outcome CIs were used as independent variables to predict subregion-specific CCT program enrollment data. All independent variables were lagged to model information that might have been available to policymakers and bureaucrats at the time of decision making for targeting the following year’s program.^4^ Subregion SES was measured in two ways to account for different frames of relative poverty – the prevalence of households in the poorest national SES quintile (absolute poverty) and the subregion’s national rank of prevalence of households in poorest SES quintile (relative poverty). Each of these variables lead to different explanatory mechanisms. If targeting the poorest populations is the primary objective, one would expect significantly higher CCT coverage in poor subregions. If targeting the sickest populations is the primary objective, one would expect significantly higher CCT coverage in subregions with worse health outcomes. Finally, the possibility that highly concentrated populations living in poverty with poor health could have given additional salience to the urgency of addressing inequalities in that subregion led to the inclusion of the CI of health inequalities as an explanatory variable.

Lastly, two predictive models were created to simulate what would have happened if policymakers had either (1) perfect information and direct control over CCT targeting (2) reasonable predictions based on information that would be available to them at the time of decision making and direct control over CCT targeting. To simulate the first counterfactual, country-specific CCT coverage was apportioned by subregion according to its share of vaccine coverage, underweight, stunting, and child deaths. To simulate the second counterfactual, a simple linear prediction based on regression of one-year lagged subregion SES rank and subregion prevalence of children and women in the poorest national SES quintile with each outcome was used to apportion country-specific CCT coverage. Both predictions do not change the number of persons enrolled in CCT programs, but rather redistribute enrollment according to each outcome and prediction method. The gap between both perfect and data-based prediction and the actual CCT program distribution was then evaluated for differences, which may be due to mistargeting or the result of some other decision-making factor.

## Results

Decompositions of generalized entropy index values into components that are explained by between-subregion components and between-SES quintile components (Table 2) reveal a generally decreasing trend in both components over time. Although this study was not designed to evaluate this trend, it appears possible that both types of inequalities have decreased at a faster rate after the introduction of CCT programs. Inequalities attributable to SES quintiles are generally larger than between-subregion inequality for most health outcomes and years, but between-subregion inequalities are always larger for vaccine coverage. Peru displays the largest between-SES quintile and between-subregion inequality for almost every measure, except for inequalities in vaccine coverage in Ecuador and SES-related inequalities in child deaths in Bolivia. In sum, both geographic and SES-related inequalities are important factors, but the relative importance varies by health outcome and country.

**Table 2:** Proportion of generalized entropy index decomposed between region (left) and between SES quintile (right) components. The higher the percentage, the greater the proportion of overall inequality that can be attributed to inequality across subregions or inequality across SES quintiles. Additional space between years indicates the point at which each country’s CCT program was first implemented.

Subregional Inequality					SES Inequality			
	DPT	Underweight	Stunting	Child Deaths	DPT	Underweight	Stunting	Child Deaths
**Bolivia**								
1998	2.13%	1.07%	3.24%	1.09%	–	–	–	–
2000	–	–	–	2.30%	–	–	–	4.15%
2003	2.24%	1.24%	4.13%	1.29%	1.22%	2.80%	8.19%	3.66%
2008	1.09%	1.67%	5.09%	1.83%	0.13%	2.34%	9.14%	3.30%
2012	–	0.41%	2.12%	–	–	1.14%	4.24%	–
**Colombia**								
2005	2.55%	1.13%	1.74%	0.43%	1.83%	1.42%	3.20%	0.72%
2010	0.85%	0.92%	1.54%	0.32%	0.50%	1.10%	1.77%	0.39%
2015	–	–	–	0.34%	–	–	–	0.46%
**Ecuador**								
1999	15.70%	–	–	1.25%	2.37%	–	–	2.02%
2004	10.79%	0.98%	4.46%	0.62%	1.13%	1.02%	3.49%	0.67%
2012	–	0.38%	2.82%	0.49%	–	0.79%	3.99%	0.71%
**Peru**								
2000	2.33%	3.96%	9.43%	3.34%	–	–	–	–
2004	0.92%	2.52%	7.46%	2.00%	0.59%	3.48%	12.32%	2.50%
2009	0.84%	3.35%	7.67%	1.88%	0.72%	4.25%	11.77%	2.14%
2010	1.50%	3.43%	6.91%	1.39%	0.20%	3.75%	10.42%	1.90%
2011	1.31%	3.41%	7.88%	2.38%	0.50%	5.08%	12.77%	2.14%
2012	0.64%	1.79%	5.66%	1.85%	0.28%	2.68%	9.15%	1.68%

In comparison to pure inequality decomposed into constituent parts, SES-related inequalities in health measured by the bivariate CI are more stable over time (Table 3). Inequalities are generally largest for the outcomes of stunting and underweight, and there is a pro-high SES distribution of DPT vaccine coverage in every survey year except for 1999 in Ecuador. Peru consistently displays the largest SES-related inequalities in health for every outcome, but also has the most progressively means-targeted (pro-low SES) distribution of CCT coverage. Importantly, Colombia, Ecuador, and Peru have CCT coverage CIs that are larger than the health inequalities they are targeting, leaving Bolivia as the only country that could not reach sufficient progressivity even with hypothetically perfect targeting.

**Table 3:** Concentration indices for each health outcome and CCT coverage CI for each country-year available. Additional space between years indicates the point at which each country’s CCT program was first implemented

	DPT	Underweight	Stunting	Child Deaths	CCT Coverage
**Bolivia**					
1998	–	–	–	–	
2000	–	–	–	−0.28	
2003	0.11	−0.37	−0.38	− 0.24	
2008	0.03	−0.37	−0.43	− 0.25	
2012	–	− 0.26	− 0.30	–	− 0.07
**Colombia**					
2005	0.17	−0.28	− 0.33	− 0.20	
2010	0.08	−0.27	− 0.24	− 0.15	−0.34
2015	–	–	–	−0.19	–
**Ecuador**					
1999	−0.19	–	–	−0.24	
2004	0.09	−0.19	−0.25	− 0.14	−0.40
2012	–	−0.19	−0.26	− 0.20	−0.54
**Peru**					
2000	–	–	–	–	
2004	0.10	−0.45	−0.49	− 0.27	
2009	0.09	− 0.50	−0.50	− 0.28	− 0.65
2010	0.04	−0.45	− 0.49	− 0.27	− 0.63
2011	0.08	− 0.53	− 0.56	− 0.28	−0.72
2012	0.05	−0.41	−0.51	− 0.26	−0.71

Targeting of subregions by health status varied greatly by outcome and country (Additional file 1: Tables 1-14). Subregions with better vaccine coverage were targeted at the same (in Colombia and Ecuador) or higher (in Bolivia and Peru) rates than other subregions, although this is partially attenuated by the omitted association with poorer subregions. Underweight and stunting were targeted at very similar rates, with higher prevalence subregions receiving higher CCT enrollment in Bolivia and Peru, but not Ecuador and Colombia. Accounting for the association of stunting and underweight with SES reduces the strength of association of targeting for Peru, but not for Bolivia. Similarly, subregions with higher prevalence of child deaths are targeted at higher rates than other subregions in Bolivia and Peru, but not Ecuador and Colombia. Bolivia is the best targeted program for child death prevalence with Peru a close second, while association with poorer subregions generally attenuates this effect for Peru, but not for children’s CCT coverage in Bolivia.

Regression of country- and subregion-specific CCT enrollment data indicates that the relative SES ranking of a subregion within a country may be guiding policymakers more than the absolute prevalence of poverty in a subregion. While there was a significant association of CCT coverage with the prevalence of women and children in the poorest national quintile of household wealth in Ecuador and Peru, Table 4 demonstrates that significant association with subregion SES ranking was present in every regression iteration except among children in Colombia.^5^ Bolivia is the only country that systematically targeted subregions with higher prevalence of health outcomes of interest more often than those with lower absolute or relative SES. In contrast, Ecuador and Peru targeted subregions with lower relative and absolute SES at higher rates, while Colombia did not appear to systematically target subregions based on health outcome prevalence, poverty, or inequality. Targeting was also fairly consistent between health outcomes, with only vaccination displaying different patterns of association in Bolivia and Peru.

**Table 4:** Regression results for targeting of CCT programs among women and children in Bolivia, Colombia, Ecuador, and Peru. SES rank represents each subregion’s relative rank from richest to poorest in the country, prevalence is specific to each health outcome (underweight, stunting, DPT vaccine coverage, and child deaths), and inequality is measured by the concentration index for each health outcome

		Women’s coverage				Children’s coverage			
		Under-weight	Stunting	DPT	Child Deaths	Under-weight	Stunting	DPT	Child Deaths
Bolivia	Prevalence	0.344***	0.0576	0.0773	0.123	1.868***	0.447***	−0.121	1.006**
		(0.0970)	(0.0460)	(0.0545)	(0.114)	(0.142)	(0.0754)	(0.242)	(0.412)
	SES Rank	0.000528	0.00171	0.00426***	0.00437***	−0.00442	−0.000325	0.0161**	0.0101*
		(0.00109)	(0.00133)	(0.00115)	(0.000663)	(0.00411)	(0.00318)	(0.00635)	(0.00474)
	Inequality	−0.00656	0.00259	0.0333**	0.00886	−0.0112	0.00116	−0.115	−0.102
		(0.0112)	(0.0153)	(0.0133)	(0.0346)	(0.0235)	(0.0514)	(0.183)	(0.156)
	Constant	0.0199**	0.0215**	−0.0342	0.0204*	0.0817**	0.0652**	0.206	0.0444
		(0.00659)	(0.00728)	(0.0410)	(0.00980)	(0.0249)	(0.0257)	(0.169)	(0.0418)
	Obs.	18	18	9	9	18	18	9	9
	R-squared	0.568	0.359	0.877	0.843	0.753	0.490	0.590	0.720
Colombia	Prevalence	0.534	−0.858	0.262	−1.918	0.583	−1.326	0.256	−2.541
		(0.966)	(0.594)	(0.457)	(1.403)	(1.513)	(0.900)	(0.731)	(2.110)
	SES Rank	0.00209	0.00559**	0.00541*	0.00532**	−0.000488	0.00422	0.00392	0.00462
		(0.00270)	(0.00270)	(0.00267)	(0.00207)	(0.00408)	(0.00400)	(0.00400)	(0.00298)
	Inequality	0.0914	0.0459	−0.333	−0.0214	0.0366	−0.0996	− 0.488	−0.0164
		(0.127)	(0.191)	(0.230)	(0.118)	(0.195)	(0.301)	(0.366)	(0.162)
	Constant	0.192***	0.239***	−0.0346	0.198***	0.342***	0.395***	0.131	0.320***
		(0.0469)	(0.0460)	(0.378)	(0.0434)	(0.0671)	(0.0689)	(0.609)	(0.0607)
	Obs.	33	33	33	66	33	33	33	66
	R-squared	0.084	0.173	0.125	0.163	0.007	0.103	0.065	0.064
Ecuador^a^	Prevalence	−0.0941	0.0126	0.147	0.598	–	–	–	–
		(0.315)	(0.156)	(0.136)	(0.904)	–	–	–	–
	SES Rank	0.0131***	0.0124***	0.0109***	0.0128***	–	–	–	–
		(0.00318)	(0.00243)	(0.00317)	(0.00238)	–	–	–	–
	Inequality	0.0242	−0.0974	0.0548	0.118	–	–	–	–
		(0.0825)	(0.143)	(0.0946)	(0.0735)	–	–	–	–
	Constant	0.165***	0.134*	0.0444	0.152***	–	–	–	–
		(0.0382)	(0.0690)	(0.0935)	(0.0520)	–	–	–	–
	Obs.	40	42	21	42	–	–	–	–
	R-squared	0.511	0.521	0.506	0.556	–	–	–	–
Peru	Prevalence	0.00353	0.00582	0.0112***	−0.0177	−0.117	0.0936	0.152***	−0.383
		(0.0120)	(0.00409)	(0.00355)	(0.0231)	(0.171)	(0.0806)	(0.0452)	(0.284)
	SES Rank	0.000313***	0.000257***	0.000316***	0.000345***	0.00710***	0.00539***	0.00634***	0.00696***
		(5.69e-05)	(5.81e-05)	(2.91e-05)	(3.73e-05)	(0.000866)	(0.00126)	(0.000495)	(0.000514)
	Inequality	−0.00106	−0.000407	0.000488	−0.00217	−0.0259*	− 0.0143	0.00127	− 0.0499
		(0.000837)	(0.00116)	(0.00314)	(0.00203)	(0.0144)	(0.0194)	(0.0415)	(0.0397)
	Constant	−0.00215***	− 0.00205***	−0.00924***	− 0.00164*	−0.0428***	− 0.0419***	−0.138***	− 0.0340**
		(0.000516)	(0.000493)	(0.00255)	(0.000809)	(0.00891)	(0.00937)	(0.0330)	(0.0148)
	Obs.	92	96	96	100	69	72	72	75
	R-squared	0.527	0.545	0.588	0.550	0.701	0.709	0.733	0.719

Robust standard errors in parentheses

*** *p* < 0.01, ** *p* < 0.05, * *p* < 0.1

^a^ Data for children’s CCT program coverage was not available for Ecuador

**Fig. 2:**
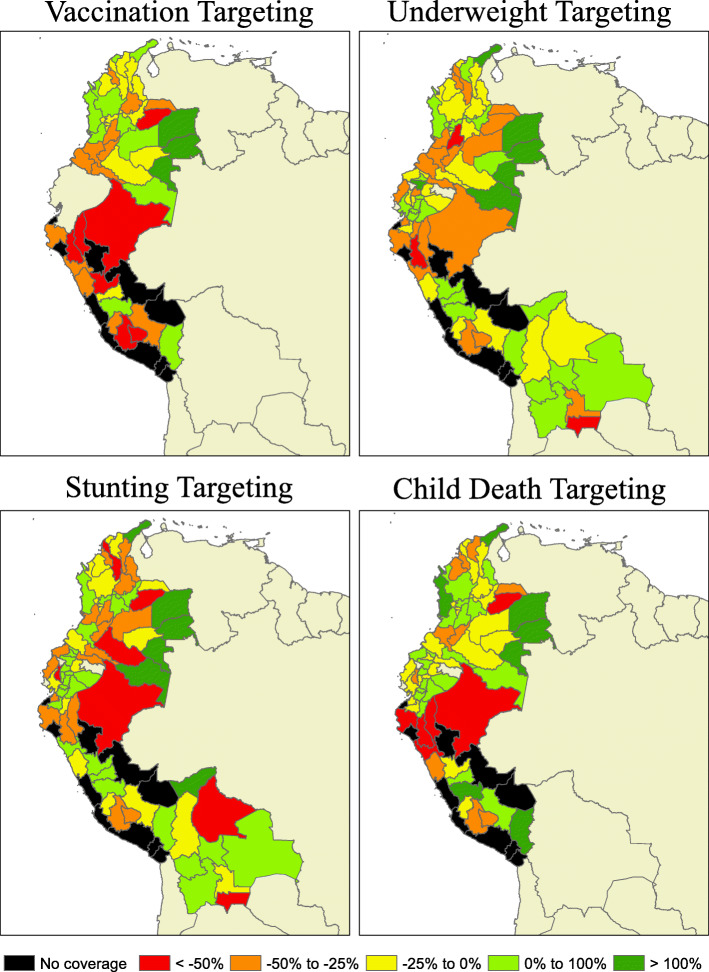
Mistargeting rates by health outcome target per subregion. Negative percentages indicate that the proportion of CCT enrollments the region received was less than the proportion of that region’s share of national prevalence of the health outcome, and positive percentages indicate the region received more CCT enrollment than its share of the health outcome

Comparing actual CCT targeting against a redistributed CCT targeting model using perfect knowledge (Fig. 2) indicates that Peru had the highest rate of mistargeting for every outcome driven by the many subregions with no CCT enrollees. Bolivia appears to have the lowest rates of mistargeting, although there are fewer data points with which to measure this efficiency (Additional file 1: Table 15). Among countries with several data points, Colombia and Ecuador have the lowest rates of mistargeting for child deaths, while Peru has the lowest rates of mistargeting for stunting. In Bolivia, Tarija is the most over-targeted subregion, and Pando the most under-targeted. In Colombia, Caldas, Cauca, Huila, Sucre, and Tolima are among the most over-targeted subregions, and Bogotá, Córdoba, La Guajira, Vaupés, and Vichada the most under-targeted. In Ecuador, Guayas, El Oro, and Manabí are among the most over-targeted subregions, and Cañar, Galápagos, and Pichincha the most under-targeted. Finally, in Peru, Áncash, Apurímac, Ayacucho, Huánuco, and Piura are among the most over-targeted subregions, and Lambayeque, Lima, San Martín, and Ucayali the most under-targeted.

The simple predictive model constructed using information available to policymakers at the time of implementation performs as well or better than the observed CCT enrollment distribution for every country, year, and outcome. While the actual CCT distribution for Bolivia is about as well targeted as the simple predictive model, Colombia and Ecuador would reduce their mistargeting by as much as half, and Peru would reduce its mistargeting by 50–75% if the simple predictive model was used.

## Discussion

The degree to which health focused CCT programs have successfully targeted the populations and health outcomes they are intended to address has yet to be quantitively evaluated using multi-country data. The underlying logic of these programs – that socioeconomic inequality and poverty is a critical determinant of health – is supported by this study’s results. Household SES was found to be a more important driver of malnutrition and mortality than geography in every country and year, regardless of the magnitude of SES-related inequalities in health. Although between-subregion inequalities in vaccine coverage are larger than SES-related inequalities, targeting by SES still greatly outperforms any subregional targeting currently used in these countries.

Using efficiency in targeting populations in need of CCT programs as an evaluation metric, universal rollout of CCT programs appears to outperform geographic targeting. Although Bolivia and Peru both targeted at-risk subregions at significantly higher rates than other subregions, subregions in Peru with no coverage result in much less efficient targeting for the country as a whole. Ecuador targets low SES populations more effectively than Colombia while maintaining universal protection, although both are mistargeting vaccine coverage, childhood malnutrition, and mortality at about the same moderate rate. Finally, and contrary to hypotheses of self-dealing by national governments through the administration of CCT programs, capital subregions were systematically under targeted in three of the four countries.

With regard to the normative frames employed by policymakers, this study provides evidence that SES – and relative rather than absolute SES – may be the dominant normative frame at work in the design of CCT program targeting. Bolivia was the sole country that appeared to target more on the basis of health outcome prevalence than on SES, which may be a direct product of policymakers’ decision to apply a weak proxy-means test rather than multidimensional household SES indicators, as other countries have done. Similarly, the higher proportion of variation in targeting explained in regression models for the process indicator of vaccine coverage than for outcome indicators of underweight and stunting may indicate that policymakers’ normative frames of access to health are outweighing those of direct health outcomes.

This study’s results are broadly consistent with previous studies of equity in targeting CCT programs in the Andean region. The relative lack of progressivity in Bolivia’s BJA targeting is supported by previous findings that although program targeting is progressive in absolute terms, over half of the program’s resources go to non-poor households [12, 56]. Colombia’s MFA program coverage was measured to have a CI of − 0.44 in 2012, a moderately higher estimate of SES-related inequality than this study’s findings, which may be due to different survey year and data source.^6^ [57] Finally, an analysis of progressivity of Latin American CCT programs using 2013 economic household survey data imply program coverage CIs of − 0.46 for Ecuador’s BDH and − 0.71 for Peru’s *Juntos* – estimates that are nearly identical to this study’s results.^7^ [58]

Although this study represents one of the most comprehensive multi-country analyses of CCT targeting to date, it did not investigate the effects of absolute CCT program coverage rates, which are significantly higher in Colombia and Ecuador. Relatedly, government agencies responsible for vaccination programs may have specifically targeted subregions known to be vulnerable to adverse childhood health outcomes, leading to issues of endemicity. The study is also limited by country-specific differences in objectives and priorities for CCT program targeting. Some countries may place more emphasis on process and access (e.g. vaccinations) and others on direct health outcomes (i.e. child mortality). Furthermore, publicly stated CCT program objectives may obscure underlying clientelist, budgetary, or logistical factors affecting program delivery. While some of these limitations can be addressed by further qualitative study, this initial quantitative evidence of the cognitive frames used by policymakers in four Andean countries provides a starting point for further study.

Two key policy implications can be reached from this analysis of targeting of CCT programs in Colombia, Ecuador, Peru, and Bolivia. First, geographic targeting may be a low-cost method of rationing scarce resources, but it is not a replacement for means-testing if CCT programs are meant to be targeted to populations with the highest levels of ill health. In fact, even a simple predictive model based on subregion-specific SES performs much better at targeting at-risk populations than any current method being used by each of these countries. Second, every CCT program in this study has achieved progressivity in targeting and are therefore successfully promoting greater health equity. Importantly, both Peru and Ecuador have targeted programs to their poorest populations effectively, demonstrating that this is possible with both universal and geographic targeting approaches. Finally, if solidarity with the poorest and sickest populations in a country is the fundamental feature of CCT programs, excluding the most vulnerable women and children that happen to live in more affluent subregions directly undermines that goal.

## Supplementary information

**Additional file 1.**

## Data Availability

All datasets analyzed are publicly available for download through the DHS Program (https://dhsprogram.com/data/available-datasets.cfm), the Unidad de Análisis de Políticas Sociales y Económicas (http://www.udape.gob.bo/index.php?option=com_wrapper&view=wrapper&Itemid=137), Global Health Data Exchange (http://ghdx.healthdata.org/record/ecuador-reproductive-health-survey-2004), the Ministerio de Salud Pública (https://www.salud.gob.ec/encuesta-nacional-de-salud-y-nutricion-ensanut/), and UNICEF (http://mics.unicef.org/surveys).

## Abbreviations

BDH: Bono de Desarrollo Humano
BJA: Bono Juana Azurduy
CCT: Conditional Cash Transfer
CI: Concentration index
DPT: Diphtheria, pertussis, and tetanus
DHS: Demographic and Health Survey
ENDEMAIN: Encuesta demográfica y de salud materna e infantil
ENSANUT: Encuesta Nacional de Salud y Nutrición
ESNUT: Encuesta de Evaluación de Salud y Nutrición
MFA: Más Familias en Acción
SES: Socioeconomic status

## Footnotes

1 The 2000 Colombia DHS is not representative at subregion level.

2 Births attended by skilled staff or deliveries occurring in health facilities could not be used because data are not comparable across countries and are dependent on preexisting infrastructure.

3 Wasting was not included as an indicator because of low prevalence at the subregion level.

4 Each regression was run separately for children’s and women’s CCT coverage.

5 Full regressions for every country, coverage population, and SES measure available in Appendix Tables 1–14

6 Author’s own calculations from Table 2. Cobertura del programa Familias en Acción a nivel de hogar por nivel de ingreso y zona in Elisa Flórez C, Rodríguez K. Subsidios sociales, pobreza y desigualdad. Departamento Nacional de Planeación; 2016 Dec p. 16–33. (Observatorio de Familia). Report No.: 8.

7 Author’s own calculations from Table 3. Distribution of Beneficiaries by Income Decile in Amarante V, Brun M. Cash Transfers in Latin America: Effects on Poverty and Redistribution. Economía. 2018;19(1):1–31.
