# Supplementary material for: Geographic Targeting and Normative Frames: Revisiting the Equity of Conditional Cash Transfer Program Distribution in Bolivia, Colombia, Ecuador, and Peru
Source: Int J Equity Health. 2020 Jul 31;19:125. doi: 10.1186/s12939-020-01233-0 (PMC7393867; doi:10.1186/s12939-020-01233-0)
Supplement: Supplementary file 1 — Additional file 1. [file 12939_2020_1233_MOESM1_ESM.docx]

Appendix

Table 1. Full regression results for targeting of Bono Juana Azurduy among Bolivian women. SES rank represents each subregion’s relative rank from richest to poorest in the country, prevalence is specific to each health outcome (underweight, stunting, DPT vaccine coverage, and child deaths), and inequality is measured by the concentration index specific to each health outcome.

| VARIABLES | SES | Underweight | | | Stunting | | | DPT | | | Child Deaths | | |
| --- | --- | --- | --- | --- | --- | --- | --- | --- | --- | --- | --- | --- | --- |
| Prevalence |  | 0.364*** | 0.336** | 0.344*** | 0.0899** | 0.0579 | 0.0576 | 0.165** | 0.0674 | 0.0773 | 0.436** | 0.115 | 0.123 |
|  |  | (0.0756) | (0.101) | (0.0970) | (0.0343) | (0.0448) | (0.0460) | (0.0566) | (0.0478) | (0.0545) | (0.176) | (0.109) | (0.114) |
| SES Rank | 0.00284** |  | 0.000472 | 0.000528 |  | 0.00169 | 0.00171 |  | 0.00459*** | 0.00426*** |  | 0.00444*** | 0.00437*** |
|  | (0.000939) |  | (0.00107) | (0.00109) |  | (0.00129) | (0.00133) |  | (0.00111) | (0.00115) |  | (0.000733) | (0.000663) |
| Inequality |  |  |  | -0.00656 |  |  | 0.00259 |  |  | 0.0333** |  |  | 0.00886 |
|  |  |  |  | (0.0112) |  |  | (0.0153) |  |  | (0.0133) |  |  | (0.0346) |
| Constant | 0.0261*** | 0.0234*** | 0.0223*** | 0.0199** | 0.0229** | 0.0207** | 0.0215** | -0.0806 | -0.0267 | -0.0342 | 0.0180 | 0.0189* | 0.0204* |
|  | (0.00462) | (0.00440) | (0.00524) | (0.00659) | (0.00714) | (0.00632) | (0.00728) | (0.0441) | (0.0355) | (0.0410) | (0.0132) | (0.00822) | (0.00980) |
| Observations | 18 | 18 | 18 | 18 | 18 | 18 | 18 | 9 | 9 | 9 | 9 | 9 | 9 |
| R-squared | 0.282 | 0.552 | 0.556 | 0.568 | 0.296 | 0.359 | 0.359 | 0.246 | 0.859 | 0.877 | 0.411 | 0.842 | 0.843 |
| Robust standard errors in parentheses *** p<0.01, ** p<0.05, * p<0.1 | | |  |  |  |  |  |  |  |  |  |  |  |

Table 2. Full regression results for targeting of Más Familias en Acción among Colombian women. SES rank represents each subregion’s relative rank from richest to poorest in the country, prevalence is specific to each health outcome (underweight, stunting, DPT vaccine coverage, and child deaths), and inequality is measured by the concentration index specific to each health outcome.

| VARIABLES | SES | Underweight | | | Stunting | | | DPT | | | Child Deaths | | |
| --- | --- | --- | --- | --- | --- | --- | --- | --- | --- | --- | --- | --- | --- |
| Prevalence |  | 0.983 | 0.505 | 0.534 | -0.269 | -0.908* | -0.858 | -0.0884 | 0.298 | 0.262 | 1.092 | -1.903 | -1.918 |
|  |  | (0.875) | (1.063) | (0.966) | (0.403) | (0.498) | (0.594) | (0.409) | (0.440) | (0.457) | (1.534) | (1.417) | (1.403) |
| SES Rank | 0.00436** |  | 0.00195 | 0.00209 |  | 0.00560** | 0.00559** |  | 0.00354 | 0.00541* |  | 0.00534** | 0.00532** |
|  | (0.00190) |  | (0.00280) | (0.00270) |  | (0.00262) | (0.00270) |  | (0.00236) | (0.00267) |  | (0.00205) | (0.00207) |
| Inequality |  |  |  | 0.0914 |  |  | 0.0459 |  |  | -0.333 |  |  | -0.0214 |
|  |  |  |  | (0.127) |  |  | (0.191) |  |  | (0.230) |  |  | (0.118) |
| Constant | 0.170*** | 0.181*** | 0.174*** | 0.192*** | 0.262*** | 0.232*** | 0.239*** | 0.303 | -0.0584 | -0.0346 | 0.217*** | 0.200*** | 0.198*** |
|  | (0.0316) | (0.0477) | (0.0458) | (0.0469) | (0.0454) | (0.0431) | (0.0460) | (0.323) | (0.363) | (0.378) | (0.0449) | (0.0387) | (0.0434) |
| Observations | 66 | 33 | 33 | 33 | 33 | 33 | 33 | 33 | 33 | 33 | 66 | 66 | 66 |
| R-squared | 0.145 | 0.045 | 0.064 | 0.084 | 0.016 | 0.170 | 0.173 | 0.002 | 0.069 | 0.125 | 0.008 | 0.163 | 0.163 |
| Robust standard errors in parentheses | | | |  |  |  |  |  |  |  |  |  |  |
| *** p<0.01, ** p<0.05, * p<0.1 | | |  |  |  |  |  |  |  |  |  |  |  |

Table 3. Full regression results for targeting of Bono de Desarrollo Humano among Ecuadorean women. SES rank represents each subregion’s relative rank from richest to poorest in the country, prevalence is specific to each health outcome (underweight, stunting, DPT vaccine coverage, and child deaths), and inequality is measured by the concentration index specific to each health outcome.

|  | SES | Underweight | | | Stunting | | | DPT | | | Child Deaths | | |
| --- | --- | --- | --- | --- | --- | --- | --- | --- | --- | --- | --- | --- | --- |
| Prevalence |  | 0.718* | -0.0519 | -0.0941 | 0.274 | 0.0257 | 0.0126 | 0.130 | 0.167 | 0.147 | 2.379* | 1.193 | 0.598 |
|  |  | (0.348) | (0.295) | (0.315) | (0.250) | (0.152) | (0.156) | (0.201) | (0.131) | (0.136) | (1.307) | (0.804) | (0.904) |
| SES Rank | 0.0124*** |  | 0.0125*** | 0.0131*** |  | 0.0123*** | 0.0124*** |  | 0.0112*** | 0.0109*** |  | 0.0118*** | 0.0128*** |
|  | (0.00265) |  | (0.00293) | (0.00318) |  | (0.00245) | (0.00243) |  | (0.00301) | (0.00317) |  | (0.00233) | (0.00238) |
| Inequality |  |  |  | 0.0242 |  |  | -0.0974 |  |  | 0.0548 |  |  | 0.118 |
|  |  |  |  | (0.0825) |  |  | (0.143) |  |  | (0.0946) |  |  | (0.0735) |
| Constant | 0.161*** | 0.236*** | 0.164*** | 0.165*** | 0.223*** | 0.155** | 0.134* | 0.189 | 0.0363 | 0.0444 | 0.215*** | 0.126** | 0.152*** |
|  | (0.0353) | (0.0404) | (0.0372) | (0.0382) | (0.0779) | (0.0571) | (0.0690) | (0.156) | (0.0945) | (0.0935) | (0.0578) | (0.0487) | (0.0520) |
| Observations | 42 | 42 | 42 | 40 | 42 | 42 | 42 | 21 | 21 | 21 | 42 | 42 | 42 |
| R-squared | 0.511 | 0.072 | 0.512 | 0.511 | 0.065 | 0.512 | 0.521 | 0.022 | 0.498 | 0.506 | 0.095 | 0.534 | 0.556 |
| Robust standard errors in parentheses | | | |  |  |  |  |  |  |  |  |  |  |
| *** p<0.01, ** p<0.05, * p<0.1 | | |  |  |  |  |  |  |  |  |  |  |  |

Table 4. Full regression results for targeting of Juntos among Peruvian women. SES rank represents each subregion’s relative rank from richest to poorest in the country, prevalence is specific to each health outcome (underweight, stunting, DPT vaccine coverage, and child deaths), and inequality is measured by the concentration index specific to each health outcome.

|  | SES | Underweight | | | Stunting | | | | | DPT | | | | Child Deaths | | | | |
| --- | --- | --- | --- | --- | --- | --- | --- | --- | --- | --- | --- | --- | --- | --- | --- | --- | --- | --- |
| Prevalence |  | 0.0418*** | 0.00405 | 0.00353 | | 0.0207*** | 0.00568 | | 0.00582 | | 0.0155** | 0.0109*** | 0.0112*** | | 0.0942*** | | -0.0222 | -0.0177 |
|  |  | (0.00805) | (0.0117) | (0.0120) | | (0.00177) | (0.00401) | | (0.00409) | | (0.00590) | (0.00292) | (0.00355) | | (0.0272) | | (0.0235) | (0.0231) |
| SES Rank | 0.000316*** |  | 0.000310*** | 0.000313*** | |  | 0.000257*** | | 0.000257*** | |  | 0.000316*** | 0.000316*** | |  | | 0.000348*** | 0.000345*** |
|  | (3.02e-05) |  | (5.75e-05) | (5.69e-05) | |  | (5.74e-05) | | (5.81e-05) | |  | (2.91e-05) | (2.91e-05) | |  | | (3.95e-05) | (3.73e-05) |
| Inequality |  |  |  | -0.00106 | |  |  | | -0.000407 | |  |  | 0.000488 | |  | |  | -0.00217 |
|  |  |  |  | (0.000837) | |  |  | | (0.00116) | |  |  | (0.00314) | |  | |  | (0.00203) |
| Constant | -0.00161*** | -0.000301 | -0.00187*** | -0.00215*** | | -0.00110*** | -0.00189*** | | -0.00205*** | | -0.00782** | -0.00901*** | -0.00924*** | | -0.00126 | | -0.00114* | -0.00164* |
|  | (0.000356) | (0.000417) | (0.000356) | (0.000516) | | (0.000264) | (0.000342) | | (0.000493) | | (0.00378) | (0.00196) | (0.00255) | | (0.00100) | | (0.000660) | (0.000809) |
| Obs. | 100 | 96 | 96 | 92 | | 96 | 96 | | 96 | | 96 | 96 | 96 | | 100 | | 100 | 100 |
| R-squared | 0.540 | 0.343 | 0.537 | 0.527 | | 0.461 | 0.544 | | 0.545 | | 0.107 | 0.587 | 0.588 | | 0.207 | | 0.546 | 0.550 |
| Robust standard errors in parentheses | | |  |  |  | |  |  | |  | |  |  |  | |  | |  |
| *** p<0.01, ** p<0.05, * p<0.1 | | |  |  |  | |  |  | |  | |  |  |  | |  | |  |

Table 5. Full regression results for targeting of Bono Juana Azurduy among Bolivian children. SES rank represents each subregion’s relative rank from richest to poorest in the country, prevalence is specific to each health outcome (underweight, stunting, DPT vaccine coverage, and child deaths), and inequality is measured by the concentration index specific to each health outcome.

|  | SES | Underweight | | | Stunting | | | DPT | | | Child Deaths | | |
| --- | --- | --- | --- | --- | --- | --- | --- | --- | --- | --- | --- | --- | --- |
| Prevalence |  | 1.586*** | 1.853*** | 1.868*** | 0.441*** | 0.447*** | 0.447*** | 0.232 | -0.0863 | -0.121 | 1.775*** | 1.102** | 1.006** |
|  |  | (0.175) | (0.146) | (0.142) | (0.0618) | (0.0704) | (0.0754) | (0.228) | (0.203) | (0.242) | (0.410) | (0.468) | (0.412) |
| SES Rank | 0.00853** |  | -0.00452 | -0.00442 |  | -0.000333 | -0.000325 |  | 0.0150** | 0.0161** |  | 0.00932 | 0.0101* |
|  | (0.00322) |  | (0.00387) | (0.00411) |  | (0.00296) | (0.00318) |  | (0.00589) | (0.00635) |  | (0.00548) | (0.00474) |
| Inequality |  |  |  | -0.0112 |  |  | 0.00116 |  |  | -0.115 |  |  | -0.102 |
|  |  |  |  | (0.0235) |  |  | (0.0514) |  |  | (0.183) |  |  | (0.156) |
| Constant | 0.107*** | 0.0758*** | 0.0859*** | 0.0817** | 0.0644*** | 0.0649** | 0.0652** | 0.00378 | 0.180 | 0.206 | 0.0592 | 0.0610* | 0.0444 |
|  | (0.0176) | (0.0138) | (0.0197) | (0.0249) | (0.0169) | (0.0194) | (0.0257) | (0.180) | (0.138) | (0.169) | (0.0371) | (0.0291) | (0.0418) |
| Observations | 18 | 18 | 18 | 18 | 18 | 18 | 18 | 9 | 9 | 9 | 9 | 9 | 9 |
| R-squared | 0.176 | 0.722 | 0.751 | 0.753 | 0.490 | 0.490 | 0.490 | 0.040 | 0.572 | 0.590 | 0.554 | 0.708 | 0.720 |
| Robust standard errors in parentheses | | | |  |  |  |  |  |  |  |  |  |  |
| *** p<0.01, ** p<0.05, * p<0.1 | | |  |  |  |  |  |  |  |  |  |  |  |

Table 6. Full regression results for targeting of Más Familias en Acción among Colombian children. SES rank represents each subregion’s relative rank from richest to poorest in the country, prevalence is specific to each health outcome (underweight, stunting, DPT vaccine coverage, and child deaths), and inequality is measured by the concentration index specific to each health outcome.

|  | SES | Underweight | | | Stunting | | | DPT | | | Child Deaths | | |
| --- | --- | --- | --- | --- | --- | --- | --- | --- | --- | --- | --- | --- | --- |
| Prevalence |  | 0.437 | 0.571 | 0.583 | -0.738 | -1.217 | -1.326 | 0.180 | 0.308 | 0.256 | 0.0727 | -2.530 | -2.541 |
|  |  | (1.163) | (1.517) | (1.513) | (0.562) | (0.719) | (0.900) | (0.605) | (0.711) | (0.731) | (2.043) | (2.124) | (2.110) |
| SES Rank | 0.00333 |  | -0.000546 | -0.000488 |  | 0.00420 | 0.00422 |  | 0.00118 | 0.00392 |  | 0.00464 | 0.00462 |
|  | (0.00265) |  | (0.00410) | (0.00408) |  | (0.00399) | (0.00400) |  | (0.00378) | (0.00400) |  | (0.00295) | (0.00298) |
| Inequality |  |  |  | 0.0366 |  |  | -0.0996 |  |  | -0.488 |  |  | -0.0164 |
|  |  |  |  | (0.195) |  |  | (0.301) |  |  | (0.366) |  |  | (0.162) |
| Constant | 0.282*** | 0.333*** | 0.335*** | 0.342*** | 0.432*** | 0.410*** | 0.395*** | 0.216 | 0.0959 | 0.131 | 0.337*** | 0.322*** | 0.320*** |
|  | (0.0430) | (0.0652) | (0.0675) | (0.0671) | (0.0635) | (0.0629) | (0.0689) | (0.482) | (0.596) | (0.609) | (0.0588) | (0.0534) | (0.0607) |
| Observations | 66 | 33 | 33 | 33 | 33 | 33 | 33 | 33 | 33 | 33 | 66 | 66 | 66 |
| R-squared | 0.047 | 0.004 | 0.005 | 0.007 | 0.057 | 0.098 | 0.103 | 0.003 | 0.007 | 0.065 | 0.000 | 0.064 | 0.064 |
| Robust standard errors in parentheses | | | |  |  |  |  |  |  |  |  |  |  |
| *** p<0.01, ** p<0.05, * p<0.1 | | |  |  |  |  |  |  |  |  |  |  |  |

Table 7. Full regression results for targeting of Juntos among Peruvian children. SES rank represents each subregion’s relative rank from richest to poorest in the country, prevalence is specific to each health outcome (underweight, stunting, DPT vaccine coverage, and child deaths), and inequality is measured by the concentration index specific to each health outcome.

|  | SES | Underweight | | | Stunting | | | DPT | | | Child Deaths | | |
| --- | --- | --- | --- | --- | --- | --- | --- | --- | --- | --- | --- | --- | --- |
| Prevalence |  | 0.727*** | -0.105 | -0.117 | 0.414*** | 0.0899 | 0.0936 | 0.245** | 0.152*** | 0.152*** | 1.724*** | -0.492* | -0.383 |
|  |  | (0.148) | (0.172) | (0.171) | (0.0502) | (0.0804) | (0.0806) | (0.103) | (0.0438) | (0.0452) | (0.426) | (0.255) | (0.284) |
| SES Rank | 0.00625*** |  | 0.00704*** | 0.00710*** |  | 0.00540*** | 0.00539*** |  | 0.00633*** | 0.00634*** |  | 0.00696*** | 0.00696*** |
|  | (0.000477) |  | (0.000897) | (0.000866) |  | (0.00126) | (0.00126) |  | (0.000489) | (0.000495) |  | (0.000517) | (0.000514) |
| Inequality |  |  |  | -0.0259* |  |  | -0.0143 |  |  | 0.00127 |  |  | -0.0499 |
|  |  |  |  | (0.0144) |  |  | (0.0194) |  |  | (0.0415) |  |  | (0.0397) |
| Constant | -0.0316*** | 0.00106 | -0.0360*** | -0.0428*** | -0.0192** | -0.0366*** | -0.0419*** | -0.116 | -0.138*** | -0.138*** | -0.0180 | -0.0215** | -0.0340** |
|  | (0.00684) | (0.00890) | (0.00689) | (0.00891) | (0.00728) | (0.00665) | (0.00937) | (0.0688) | (0.0309) | (0.0330) | (0.0174) | (0.00921) | (0.0148) |
| Observations | 75 | 72 | 72 | 69 | 72 | 72 | 72 | 72 | 72 | 72 | 75 | 75 | 75 |
| R-squared | 0.701 | 0.358 | 0.704 | 0.701 | 0.589 | 0.707 | 0.709 | 0.086 | 0.733 | 0.733 | 0.241 | 0.711 | 0.719 |
| Robust standard errors in parentheses | | | |  |  |  |  |  |  |  |  |  |  |
| *** p<0.01, ** p<0.05, * p<0.1 | | |  |  |  |  |  |  |  |  |  |  |  |

Table 8. Full regression results for targeting of Bono Juana Azurduy among Bolivian women. Poverty represents each subregion’s absolute prevalence of households in the poorest national quintile, prevalence is specific to each health outcome (underweight, stunting, DPT vaccine coverage, and child deaths), and inequality is measured by the concentration index specific to each health outcome.

|  | Poverty | Underweight | | | Stunting | | | DPT | | | Child Deaths | | |
| --- | --- | --- | --- | --- | --- | --- | --- | --- | --- | --- | --- | --- | --- |
| Prevalence |  | 0.364*** | 0.369*** | 0.379*** | 0.0899** | 0.0889* | 0.0889* | 0.165** | 0.0753*** | 0.0740*** | 0.436** | -0.257*** | -0.257*** |
|  |  | (0.0756) | (0.0810) | (0.0747) | (0.0343) | (0.0425) | (0.0439) | (0.0566) | (0.0148) | (0.0152) | (0.176) | (0.0253) | (0.0401) |
| Poverty | 0.0506 |  | -0.00407 | -0.00336 |  | 0.00185 | 0.00180 |  | 0.169*** | 0.171*** |  | 0.239*** | 0.239*** |
|  | (0.0363) |  | (0.0192) | (0.0206) |  | (0.0265) | (0.0277) |  | (0.0105) | (0.0128) |  | (0.00946) | (0.0110) |
| Inequality |  |  |  | -0.00618 |  |  | -0.00153 |  |  | -0.00402 |  |  | -0.000249 |
|  |  |  |  | (0.0109) |  |  | (0.0185) |  |  | (0.0174) |  |  | (0.0125) |
| Constant | 0.0301*** | 0.0234*** | 0.0239*** | 0.0217** | 0.0229** | 0.0228** | 0.0223** | -0.0806 | -0.0395*** | -0.0386** | 0.0180 | 0.0262*** | 0.0262*** |
|  | (0.00702) | (0.00440) | (0.00607) | (0.00755) | (0.00714) | (0.00721) | (0.00763) | (0.0441) | (0.0117) | (0.0121) | (0.0132) | (0.00233) | (0.00215) |
| Observations | 18 | 18 | 18 | 18 | 18 | 18 | 18 | 9 | 9 | 9 | 9 | 9 | 9 |
| R-squared | 0.097 | 0.552 | 0.552 | 0.563 | 0.296 | 0.296 | 0.297 | 0.246 | 0.981 | 0.981 | 0.411 | 0.985 | 0.985 |
| Robust standard errors in parentheses | | |  |  |  |  |  |  |  |  |  |  |  |
| *** p<0.01, ** p<0.05, *p<0.1 | |  |  |  |  |  |  |  |  |  |  |  |  |

Table 9. Full regression results for targeting of Más Familias en Acción among Colombian women. Poverty represents each subregion’s absolute prevalence of households in the poorest national quintile, prevalence is specific to each health outcome (underweight, stunting, DPT vaccine coverage, and child deaths), and inequality is measured by the concentration index specific to each health outcome.

|  | Poverty | Underweight | | | Stunting | | | DPT | | | Child Deaths | | |
| --- | --- | --- | --- | --- | --- | --- | --- | --- | --- | --- | --- | --- | --- |
| Prevalence |  | 0.983 | 1.065 | 1.112 | -0.269 | -0.726 | -0.675 | -0.0884 | 0.0471 | 0.0402 | 1.092 | -0.905 | -0.921 |
|  |  | (0.875) | (1.147) | (1.058) | (0.403) | (0.538) | (0.622) | (0.409) | (0.472) | (0.490) | (1.534) | (1.734) | (1.710) |
| Poverty | 0.129 |  | -0.0176 | -0.0149 |  | 0.187 | 0.186 |  | 0.0658 | 0.152 |  | 0.155 | 0.152 |
|  | (0.104) |  | (0.142) | (0.139) |  | (0.145) | (0.150) |  | (0.129) | (0.172) |  | (0.125) | (0.124) |
| Inequality |  |  |  | 0.0843 |  |  | 0.0463 |  |  | -0.258 |  |  | -0.0351 |
|  |  |  |  | (0.121) |  |  | (0.194) |  |  | (0.284) |  |  | (0.133) |
| Constant | 0.208*** | 0.181*** | 0.182*** | 0.199*** | 0.262*** | 0.256*** | 0.263*** | 0.303 | 0.179 | 0.181 | 0.217*** | 0.223*** | 0.219*** |
|  | (0.0311) | (0.0477) | (0.0489) | (0.0513) | (0.0454) | (0.0443) | (0.0478) | (0.323) | (0.389) | (0.406) | (0.0449) | (0.0410) | (0.0459) |
| Observations | 66 | 33 | 33 | 33 | 33 | 33 | 33 | 33 | 33 | 33 | 66 | 66 | 66 |
| R-squared | 0.054 | 0.045 | 0.046 | 0.063 | 0.016 | 0.076 | 0.079 | 0.002 | 0.011 | 0.040 | 0.008 | 0.058 | 0.059 |
| Robust standard errors in parentheses | | |  |  |  |  |  |  |  |  |  |  |  |
| *** p<0.01, ** p<0.05, * p<0.1 | | |  |  |  |  |  |  |  |  |  |  |  |

Table 10. Full regression results for targeting of Bono de Desarrollo Humano among Ecuadorean women. Poverty represents each subregion’s absolute prevalence of households in the poorest national quintile, prevalence is specific to each health outcome (underweight, stunting, DPT vaccine coverage, and child deaths), and inequality is measured by the concentration index specific to each health outcome.

|  | Poverty | Underweight | | | Stunting | | | DPT | | | Child Deaths | | |
| --- | --- | --- | --- | --- | --- | --- | --- | --- | --- | --- | --- | --- | --- |
| Prevalence |  | 0.718* | -0.200 | -0.237 | 0.274 | -0.0369 | -0.0399 | 0.130 | 0.153 | 0.144 | 2.379* | 0.835 | 0.249 |
|  |  | (0.348) | (0.246) | (0.265) | (0.250) | (0.153) | (0.159) | (0.201) | (0.120) | (0.124) | (1.307) | (0.722) | (0.874) |
| Poverty | 0.704*** |  | 0.734*** | 0.754*** |  | 0.716*** | 0.715*** |  | 0.591*** | 0.584*** |  | 0.676*** | 0.723*** |
|  | (0.114) |  | (0.130) | (0.139) |  | (0.115) | (0.116) |  | (0.111) | (0.125) |  | (0.102) | (0.0988) |
| Inequality |  |  |  | 0.0313 |  |  | -0.0307 |  |  | 0.0249 |  |  | 0.114 |
|  |  |  |  | (0.0810) |  |  | (0.128) |  |  | (0.0824) |  |  | (0.0731) |
| Constant | 0.145*** | 0.236*** | 0.156*** | 0.159*** | 0.223*** | 0.153*** | 0.146** | 0.189 | 0.0456 | 0.0494 | 0.215*** | 0.122** | 0.148** |
|  | (0.0334) | (0.0404) | (0.0330) | (0.0335) | (0.0779) | (0.0517) | (0.0603) | (0.156) | (0.0831) | (0.0846) | (0.0578) | (0.0458) | (0.0520) |
| Observations | 42 | 42 | 42 | 40 | 42 | 42 | 42 | 21 | 21 | 21 | 42 | 42 | 42 |
| R-squared | 0.576 | 0.072 | 0.581 | 0.582 | 0.065 | 0.577 | 0.578 | 0.022 | 0.600 | 0.602 | 0.095 | 0.587 | 0.608 |
| Robust standard errors in parentheses | | |  |  |  |  |  |  |  |  |  |  |  |
| *** p<0.01, ** p<0.05, * p<0.1 | | |  |  |  |  |  |  |  |  |  |  |  |

Table 11. Full regression results for targeting of Juntos among Peruvian women. Poverty represents each subregion’s absolute prevalence of households in the poorest national quintile, prevalence is specific to each health outcome (underweight, stunting, DPT vaccine coverage, and child deaths), and inequality is measured by the concentration index specific to each health outcome.

|  | Poverty | Underweight | | | Stunting | | | DPT | | | Child Deaths | | |
| --- | --- | --- | --- | --- | --- | --- | --- | --- | --- | --- | --- | --- | --- |
| Prevalence |  | 0.0418*** | -0.00161 | -0.00182 | 0.0207*** | 0.00118 | 0.00132 | 0.0155** | 0.00962*** | 0.00990*** | 0.0942*** | -0.0219 | -0.0171 |
|  |  | (0.00805) | (0.00954) | (0.00960) | (0.00177) | (0.00302) | (0.00308) | (0.00590) | (0.00248) | (0.00323) | (0.0272) | (0.0190) | (0.0184) |
| Poverty | 0.0143*** |  | 0.0148*** | 0.0149*** |  | 0.0139*** | 0.0139*** |  | 0.0139*** | 0.0139*** |  | 0.0156*** | 0.0155*** |
|  | (0.00102) |  | (0.00211) | (0.00209) |  | (0.00178) | (0.00179) |  | (0.00108) | (0.00106) |  | (0.00164) | (0.00155) |
| Inequality |  |  |  | -0.00119 |  |  | -0.000731 |  |  | 0.000505 |  |  | -0.00256 |
|  |  |  |  | (0.000752) |  |  | (0.00115) |  |  | (0.00267) |  |  | (0.00192) |
| Constant | -0.000463** | -0.000301 | -0.000469 | -0.000811** | -0.00110*** | -0.000591* | -0.000868 | -0.00782** | -0.00686*** | -0.00709*** | -0.00126 | 0.000150 | -0.000440 |
|  | (0.000224) | (0.000417) | (0.000303) | (0.000384) | (0.000264) | (0.000325) | (0.000511) | (0.00378) | (0.00164) | (0.00231) | (0.00100) | (0.000543) | (0.000630) |
| Observations | 100 | 96 | 96 | 92 | 96 | 96 | 96 | 96 | 96 | 96 | 100 | 100 | 100 |
| R-squared | 0.621 | 0.343 | 0.612 | 0.604 | 0.461 | 0.612 | 0.613 | 0.107 | 0.652 | 0.652 | 0.207 | 0.627 | 0.634 |
| Robust standard errors in parentheses | | |  |  |  |  |  |  |  |  |  |  |  |
| *** p<0.01, ** p<0.05, * p<0.1 | |  |  |  |  |  |  |  |  |  |  |  |  |

Table 12. Full regression results for targeting of Bono Juana Azurduy among Bolivian women. Poverty represents each subregion’s absolute prevalence of households in the poorest national quintile, prevalence is specific to each health outcome (underweight, stunting, DPT vaccine coverage, and child deaths), and inequality is measured by the concentration index specific to each health outcome.

|  | Poverty | Underweight | | | Stunting | | | DPT | | | Child Deaths | | |
| --- | --- | --- | --- | --- | --- | --- | --- | --- | --- | --- | --- | --- | --- |
| Prevalence |  | 1.586*** | 1.779*** | 1.799*** | 0.441*** | 0.540*** | 0.540*** | 0.232 | -0.0555 | -0.141 | 1.775*** | 0.636 | 0.502 |
|  |  | (0.175) | (0.177) | (0.180) | (0.0618) | (0.0933) | (0.0969) | (0.228) | (0.172) | (0.249) | (0.410) | (0.735) | (0.787) |
| Poverty | 0.120 |  | -0.143 | -0.142 |  | -0.176* | -0.176* |  | 0.543*** | 0.659*** |  | 0.393 | 0.427 |
|  | (0.133) |  | (0.101) | (0.107) |  | (0.0778) | (0.0816) |  | (0.0909) | (0.130) |  | (0.220) | (0.235) |
| Inequality |  |  |  | -0.0122 |  |  | -0.000659 |  |  | -0.263 |  |  | -0.0916 |
|  |  |  |  | (0.0194) |  |  | (0.0530) |  |  | (0.208) |  |  | (0.224) |
| Constant | 0.125*** | 0.0758*** | 0.0956*** | 0.0910*** | 0.0644*** | 0.0805*** | 0.0803** | 0.00378 | 0.136 | 0.193 | 0.0592 | 0.0727* | 0.0587 |
|  | (0.0290) | (0.0138) | (0.0230) | (0.0269) | (0.0169) | (0.0223) | (0.0271) | (0.180) | (0.129) | (0.189) | (0.0371) | (0.0316) | (0.0503) |
| Observations | 18 | 18 | 18 | 18 | 18 | 18 | 18 | 9 | 9 | 9 | 9 | 9 | 9 |
| R-squared | 0.038 | 0.722 | 0.765 | 0.768 | 0.490 | 0.545 | 0.545 | 0.040 | 0.657 | 0.735 | 0.554 | 0.680 | 0.690 |
| Robust standard errors in parentheses | | |  |  |  |  |  |  |  |  |  |  |  |
| *** p<0.01, ** p<0.05, * p<0.1 | | |  |  |  |  |  |  |  |  |  |  |  |

Table 13. Full regression results for targeting of Más Familias en Acción among Colombian children. Poverty represents each subregion’s absolute prevalence of households in the poorest national quintile, prevalence is specific to each health outcome (underweight, stunting, DPT vaccine coverage, and child deaths), and inequality is measured by the concentration index specific to each health outcome.

|  | Poverty | Underweight | | | Stunting | | | DPT | | | Child Deaths | | |
| --- | --- | --- | --- | --- | --- | --- | --- | --- | --- | --- | --- | --- | --- |
| Prevalence |  | 0.437 | 1.331 | 1.350 | -0.738 | -0.892 | -1.001 | 0.180 | -0.0188 | -0.0278 | 0.0727 | -0.837 | -0.854 |
|  |  | (1.163) | (1.630) | (1.631) | (0.562) | (0.783) | (0.936) | (0.605) | (0.720) | (0.745) | (2.043) | (2.466) | (2.449) |
| Poverty | 0.0469 |  | -0.190 | -0.189 |  | 0.0630 | 0.0647 |  | -0.0964 | 0.0150 |  | 0.0705 | 0.0675 |
|  | (0.135) |  | (0.203) | (0.205) |  | (0.220) | (0.218) |  | (0.188) | (0.243) |  | (0.166) | (0.165) |
| Inequality |  |  |  | 0.0332 |  |  | -0.0973 |  |  | -0.331 |  |  | -0.0381 |
|  |  |  |  | (0.175) |  |  | (0.299) |  |  | (0.425) |  |  | (0.176) |
| Constant | 0.325*** | 0.333*** | 0.338*** | 0.345*** | 0.432*** | 0.430*** | 0.416*** | 0.216 | 0.398 | 0.401 | 0.337*** | 0.339*** | 0.335*** |
|  | (0.0404) | (0.0652) | (0.0710) | (0.0733) | (0.0635) | (0.0637) | (0.0692) | (0.482) | (0.597) | (0.617) | (0.0588) | (0.0572) | (0.0641) |
| Observations | 66 | 33 | 33 | 33 | 33 | 33 | 33 | 33 | 33 | 33 | 66 | 66 | 66 |
| R-squared | 0.004 | 0.004 | 0.039 | 0.040 | 0.057 | 0.060 | 0.065 | 0.003 | 0.013 | 0.036 | 0.000 | 0.006 | 0.006 |
| Robust standard errors in parentheses | | |  |  |  |  |  |  |  |  |  |  |  |
| *** p<0.01, ** p<0.05, * p<0.1 | | |  |  |  |  |  |  |  |  |  |  |  |

Table 14. Full regression results for targeting of Juntos among Peruvian children. Poverty represents each subregion’s absolute prevalence of households in the poorest national quintile, prevalence is specific to each health outcome (underweight, stunting, DPT vaccine coverage, and child deaths), and inequality is measured by the concentration index specific to each health outcome.

|  | Poverty | Underweight | | | Stunting | | | DPT | | | Child Deaths | | |
| --- | --- | --- | --- | --- | --- | --- | --- | --- | --- | --- | --- | --- | --- |
| Prevalence |  | 0.727*** | -0.142 | -0.148 | 0.414*** | 0.0393 | 0.0422 | 0.245** | 0.130*** | 0.129*** | 1.724*** | -0.380** | -0.282 |
|  |  | (0.148) | (0.147) | (0.145) | (0.0502) | (0.0645) | (0.0641) | (0.103) | (0.0384) | (0.0399) | (0.426) | (0.182) | (0.189) |
| Poverty | 0.267*** |  | 0.296*** | 0.298*** |  | 0.249*** | 0.250*** |  | 0.260*** | 0.260*** |  | 0.288*** | 0.287*** |
|  | (0.0121) |  | (0.0325) | (0.0323) |  | (0.0398) | (0.0397) |  | (0.0159) | (0.0158) |  | (0.0151) | (0.0155) |
| Inequality |  |  |  | -0.0260* |  |  | -0.0211 |  |  | -0.00201 |  |  | -0.0430 |
|  |  |  |  | (0.0139) |  |  | (0.0196) |  |  | (0.0355) |  |  | (0.0363) |
| Constant | -0.00571 | 0.00106 | -0.00235 | -0.00943* | -0.0192** | -0.00878 | -0.0167* | -0.116 | -0.0937*** | -0.0929*** | -0.0180 | 0.00476 | -0.00608 |
|  | (0.00453) | (0.00890) | (0.00491) | (0.00532) | (0.00728) | (0.00541) | (0.00823) | (0.0688) | (0.0259) | (0.0279) | (0.0174) | (0.00668) | (0.0107) |
| Observations | 75 | 72 | 72 | 69 | 72 | 72 | 72 | 72 | 72 | 72 | 75 | 75 | 75 |
| R-squared | 0.751 | 0.358 | 0.748 | 0.746 | 0.589 | 0.743 | 0.746 | 0.086 | 0.766 | 0.766 | 0.241 | 0.758 | 0.764 |
| Robust standard errors in parentheses | | |  |  |  |  |  |  |  |  |  |  |  |
| *** p<0.01, ** p<0.05, * p<0.1 | | |  |  |  |  |  |  |  |  |  |  |  |

Table 15. Perfect prediction model with the actual number of CCT enrollees per subregion, the number of enrollees expected in that subregion with perfect targeting for each health outcome, and the percent deviation between the actual and expected targeting.

|  |  |  | Actual | DPT | | Underweight | | Stunting | | Child deaths | |
| --- | --- | --- | --- | --- | --- | --- | --- | --- | --- | --- | --- |
| Year | Country | Region | # | # | % ∆ | # | % ∆ | # | % ∆ | # | % ∆ |
| 2012 | Bolivia | La Paz | 19588 |  |  | 18212 | -7% | 18346 | -6% |  |  |
| 2012 | Bolivia | Santa Cruz | 8392 |  |  | 12450 | 48% | 12752 | 52% |  |  |
| 2012 | Bolivia | Tarija | 4029 |  |  | 1070 | -73% | 1492 | -63% |  |  |
| 2012 | Bolivia | Cochabamba | 10416 |  |  | 10624 | 2% | 11728 | 13% |  |  |
| 2012 | Bolivia | Oruro | 3710 |  |  | 4305 | 16% | 4540 | 22% |  |  |
| 2012 | Bolivia | Pando | 185 |  |  | 365 | 97% | 746 | 303% |  |  |
| 2012 | Bolivia | Chuquisaca | 6295 |  |  | 4341 | -31% | 4764 | -24% |  |  |
| 2012 | Bolivia | Beni | 3740 |  |  | 3385 | -9% | 1709 | -54% |  |  |
| 2012 | Bolivia | Potosí | 8371 |  |  | 9974 | 19% | 8649 | 3% |  |  |
| 2005 | Colombia | Antioquia | 66760 | 74892 | 12% | 62472 | -6% | 66118 | -1% | 87771 | 31% |
| 2005 | Colombia | Guainía | 446 | 662 | 48% | 690 | 55% | 493 | 11% | 684 | 53% |
| 2005 | Colombia | Casanare | 9312 | 5086 | -45% | 2807 | -70% | 1341 | -86% | 3380 | -64% |
| 2005 | Colombia | Santander | 25528 | 23751 | -7% | 18859 | -26% | 16686 | -35% | 20848 | -18% |
| 2005 | Colombia | Arauca | 9609 | 5023 | -48% | 5856 | -39% | 3413 | -64% | 4680 | -51% |
| 2005 | Colombia | Guaviare | 2899 | 2420 | -17% | 1693 | -42% | 946 | -67% | 2439 | -16% |
| 2005 | Colombia | Cauca | 30521 | 30314 | -1% | 27679 | -9% | 26958 | -12% | 35782 | 17% |
| 2005 | Colombia | Meta | 13177 | 12422 | -6% | 8792 | -33% | 9137 | -31% | 10839 | -18% |
| 2005 | Colombia | Bolívar | 43019 | 27188 | -37% | 49855 | 16% | 40953 | -5% | 33410 | -22% |
| 2005 | Colombia | Huila | 29784 | 18879 | -37% | 17081 | -43% | 19223 | -35% | 19218 | -35% |
| 2005 | Colombia | Córdoba | 53564 | 35969 | -33% | 45800 | -14% | 34788 | -35% | 27795 | -48% |
| 2005 | Colombia | Putumayo | 10251 | 5923 | -42% | 2439 | -76% | 4982 | -51% | 6382 | -38% |
| 2005 | Colombia | Nariño | 37832 | 17303 | -54% | 30871 | -18% | 45724 | 21% | 35180 | -7% |
| 2005 | Colombia | Chocó | 16315 | 15834 | -3% | 8962 | -45% | 6329 | -61% | 16666 | 2% |
| 2005 | Colombia | Caquetá | 14387 | 12934 | -10% | 10258 | -29% | 7501 | -48% | 13771 | -4% |
| 2005 | Colombia | Caldas | 8829 | 7975 | -10% | 10621 | 20% | 10786 | 22% | 9042 | 2% |
| 2005 | Colombia | San Andrés y Providencia | 1302 | 1093 | -16% | 636 | -51% | 149 | -89% | 663 | -49% |
| 2005 | Colombia | Vichada | 902 | 1523 | 69% | 1374 | 52% | 1206 | 34% | 1881 | 109% |
| 2005 | Colombia | Bogotá | 7640 | 84960 | 1012% | 75503 | 888% | 95482 | 1150% | 66792 | 774% |
| 2005 | Colombia | Sucre | 37458 | 15205 | -59% | 18156 | -52% | 12563 | -66% | 11365 | -70% |
| 2005 | Colombia | Atlántico | 18505 | 33060 | 79% | 33465 | 81% | 29780 | 61% | 29037 | 57% |
| 2005 | Colombia | Boyacá | 25398 | 19453 | -23% | 35219 | 39% | 39854 | 57% | 16476 | -35% |
| 2005 | Colombia | Vaupés | 292 | 754 | 158% | 1414 | 384% | 1057 | 262% | 978 | 235% |
| 2005 | Colombia | Magdalena | 32410 | 34002 | 5% | 31785 | -2% | 33441 | 3% | 27206 | -16% |
| 2005 | Colombia | Valle del Cauca | 24326 | 50763 | 109% | 43739 | 80% | 33185 | 36% | 54810 | 125% |
| 2005 | Colombia | Risaralda | 5783 | 7675 | 33% | 7443 | 29% | 6678 | 15% | 11099 | 92% |
| 2005 | Colombia | Quindío | 10614 | 3269 | -69% | 4410 | -58% | 4650 | -56% | 5345 | -50% |
| 2005 | Colombia | Amazonas | 665 | 1388 | 109% | 1833 | 176% | 1703 | 156% | 1487 | 124% |
| 2005 | Colombia | La Guajira | 11953 | 23203 | 94% | 31097 | 160% | 30454 | 155% | 22063 | 85% |
| 2005 | Colombia | Cundinamarca | 33148 | 27258 | -18% | 18104 | -45% | 26978 | -19% | 23902 | -28% |
| 2005 | Colombia | Tolima | 32703 | 25192 | -23% | 30144 | -8% | 20340 | -38% | 22827 | -30% |
| 2005 | Colombia | Cesar | 28974 | 22202 | -23% | 20832 | -28% | 16406 | -43% | 20854 | -28% |
| 2005 | Colombia | Norte de Santander | 21009 | 17742 | -16% | 5428 | -74% | 16009 | -24% | 20643 | -2% |
| 2010 | Colombia | Nariño | 128705 | 74719 | -42% | 85156 | -34% | 116474 | -10% | 103716 | -19% |
| 2010 | Colombia | Bolívar | 182870 | 155802 | -15% | 122735 | -33% | 81441 | -55% | 143595 | -21% |
| 2010 | Colombia | Córdoba | 170938 | 145135 | -15% | 143500 | -16% | 141690 | -17% | 118850 | -30% |
| 2010 | Colombia | Vichada | 2346 | 5936 | 153% | 8978 | 283% | 7099 | 203% | 4799 | 105% |
| 2010 | Colombia | Cesar | 81878 | 66603 | -19% | 74645 | -9% | 60933 | -26% | 75591 | -8% |
| 2010 | Colombia | Norte de Santander | 83932 | 73186 | -13% | 81460 | -3% | 51882 | -38% | 86417 | 3% |
| 2010 | Colombia | Valle del Cauca | 142910 | 205098 | 44% | 84121 | -41% | 87647 | -39% | 174678 | 22% |
| 2010 | Colombia | Amazonas | 3998 | 5818 | 46% | 9576 | 140% | 12926 | 223% | 7805 | 95% |
| 2010 | Colombia | Caldas | 39463 | 34276 | -13% | 43377 | 10% | 50755 | 29% | 39830 | 1% |
| 2010 | Colombia | Magdalena | 119616 | 102809 | -14% | 152167 | 27% | 119309 | 0% | 85727 | -28% |
| 2010 | Colombia | Cauca | 120416 | 68000 | -44% | 86003 | -29% | 143710 | 19% | 70422 | -42% |
| 2010 | Colombia | Guainía | 857 | 3980 | 364% | 5483 | 540% | 5758 | 572% | 3543 | 313% |
| 2010 | Colombia | Arauca | 25424 | 12952 | -49% | 18289 | -28% | 20553 | -19% | 15879 | -38% |
| 2010 | Colombia | Quindío | 26690 | 21226 | -20% | 24914 | -7% | 21951 | -18% | 24259 | -9% |
| 2010 | Colombia | San Andrés y Providencia | 1663 | 4965 | 199% | 1999 | 20% | 1408 | -15% | 3177 | 91% |
| 2010 | Colombia | Casanare | 32443 | 12130 | -63% | 18096 | -44% | 12008 | -63% | 12914 | -60% |
| 2010 | Colombia | Meta | 49266 | 51649 | 5% | 34616 | -30% | 35482 | -28% | 49169 | 0% |
| 2010 | Colombia | Antioquia | 246401 | 309976 | 26% | 218019 | -12% | 231965 | -6% | 267778 | 9% |
| 2010 | Colombia | Guaviare | 8376 | 8316 | -1% | 10096 | 21% | 7817 | -7% | 6820 | -19% |
| 2010 | Colombia | Atlántico | 140564 | 141194 | 0% | 117321 | -17% | 131144 | -7% | 125656 | -11% |
| 2010 | Colombia | Cundinamarca | 100967 | 126160 | 25% | 100577 | 0% | 127197 | 26% | 112193 | 11% |
| 2010 | Colombia | Sucre | 94085 | 55252 | -41% | 67984 | -28% | 56497 | -40% | 48713 | -48% |
| 2010 | Colombia | La Guajira | 48251 | 93208 | 93% | 203050 | 321% | 151195 | 213% | 117242 | 143% |
| 2010 | Colombia | Risaralda | 39188 | 31921 | -19% | 39209 | 0% | 37067 | -5% | 35618 | -9% |
| 2010 | Colombia | Putumayo | 32021 | 21771 | -32% | 31391 | -2% | 18041 | -44% | 36444 | 14% |
| 2010 | Colombia | Huila | 93076 | 53897 | -42% | 58144 | -38% | 50448 | -46% | 69569 | -25% |
| 2010 | Colombia | Santander | 98039 | 72910 | -26% | 95971 | -2% | 52967 | -46% | 74566 | -24% |
| 2010 | Colombia | Chocó | 32053 | 55862 | 74% | 54209 | 69% | 44321 | 38% | 89690 | 180% |
| 2010 | Colombia | Caquetá | 44468 | 34746 | -22% | 37956 | -15% | 19051 | -57% | 39624 | -11% |
| 2010 | Colombia | Bogotá | 127577 | 299869 | 135% | 327497 | 157% | 405409 | 218% | 272986 | 114% |
| 2010 | Colombia | Vaupés | 490 | 3590 | 633% | 3009 | 514% | 9474 | 1833% | 3268 | 567% |
| 2010 | Colombia | Boyacá | 71285 | 63357 | -11% | 89057 | 25% | 106552 | 49% | 69265 | -3% |
| 2010 | Colombia | Tolima | 96477 | 70420 | -27% | 38129 | -60% | 66561 | -31% | 96929 | 0% |
| 2015 | Colombia | Nariño | 155777 |  |  |  |  |  |  | 109844 | -29% |
| 2015 | Colombia | Cauca | 138329 |  |  |  |  |  |  | 79021 | -43% |
| 2015 | Colombia | Cundinamarca | 89980 |  |  |  |  |  |  | 99054 | 10% |
| 2015 | Colombia | Bogotá | 68085 |  |  |  |  |  |  | 310188 | 356% |
| 2015 | Colombia | Sucre | 92576 |  |  |  |  |  |  | 34718 | -62% |
| 2015 | Colombia | Vichada | 2320 |  |  |  |  |  |  | 6106 | 163% |
| 2015 | Colombia | Vaupés | 1092 |  |  |  |  |  |  | 10625 | 873% |
| 2015 | Colombia | Meta | 61755 |  |  |  |  |  |  | 39421 | -36% |
| 2015 | Colombia | Córdoba | 193620 |  |  |  |  |  |  | 85398 | -56% |
| 2015 | Colombia | Guainía | 2219 |  |  |  |  |  |  | 3975 | 79% |
| 2015 | Colombia | Guaviare | 8515 |  |  |  |  |  |  | 8031 | -6% |
| 2015 | Colombia | Boyacá | 62514 |  |  |  |  |  |  | 67996 | 9% |
| 2015 | Colombia | Antioquia | 296873 |  |  |  |  |  |  | 299294 | 1% |
| 2015 | Colombia | Atlántico | 116414 |  |  |  |  |  |  | 128930 | 11% |
| 2015 | Colombia | Cesar | 94867 |  |  |  |  |  |  | 78387 | -17% |
| 2015 | Colombia | Norte de Santander | 99377 |  |  |  |  |  |  | 82302 | -17% |
| 2015 | Colombia | Amazonas | 5111 |  |  |  |  |  |  | 11716 | 129% |
| 2015 | Colombia | San Andrés y Providencia | 2493 |  |  |  |  |  |  | 3575 | 43% |
| 2015 | Colombia | Arauca | 24548 |  |  |  |  |  |  | 24915 | 1% |
| 2015 | Colombia | Casanare | 33399 |  |  |  |  |  |  | 24098 | -28% |
| 2015 | Colombia | Caquetá | 45129 |  |  |  |  |  |  | 44287 | -2% |
| 2015 | Colombia | Santander | 98584 |  |  |  |  |  |  | 78709 | -20% |
| 2015 | Colombia | Magdalena | 122257 |  |  |  |  |  |  | 88312 | -28% |
| 2015 | Colombia | Risaralda | 39034 |  |  |  |  |  |  | 39315 | 1% |
| 2015 | Colombia | Huila | 96937 |  |  |  |  |  |  | 64397 | -34% |
| 2015 | Colombia | La Guajira | 61519 |  |  |  |  |  |  | 170861 | 178% |
| 2015 | Colombia | Valle del Cauca | 139072 |  |  |  |  |  |  | 217822 | 57% |
| 2015 | Colombia | Tolima | 97856 |  |  |  |  |  |  | 58411 | -40% |
| 2015 | Colombia | Quindío | 19068 |  |  |  |  |  |  | 20752 | 9% |
| 2015 | Colombia | Caldas | 41683 |  |  |  |  |  |  | 38460 | -8% |
| 2015 | Colombia | Bolívar | 169218 |  |  |  |  |  |  | 121346 | -28% |
| 2015 | Colombia | Putumayo | 37462 |  |  |  |  |  |  | 32887 | -12% |
| 2015 | Colombia | Chocó | 42271 |  |  |  |  |  |  | 76801 | 82% |
| 2004 | Ecuador | Morona Santiago | 8177 | 10198 | 25% | 6415 | -22% | 8881 | 9% | 14398 | 76% |
| 2004 | Ecuador | Pastaza | 4213 | 2862 | -32% | 3565 | -15% | 6410 | 52% | 1853 | -56% |
| 2004 | Ecuador | Napo | 14557 | 12631 | -13% | 36485 | 151% | 19194 | 32% | 9836 | -32% |
| 2004 | Ecuador | Galápagos | 247 | 350 | 42% | 1338 | 442% | 533 | 116% | 678 | 175% |
| 2004 | Ecuador | Pichincha | 89030 | 274317 | 208% | 125831 | 41% | 183194 | 106% | 129253 | 45% |
| 2004 | Ecuador | Loja | 32411 | 35690 | 10% | 45692 | 41% | 33971 | 5% | 31582 | -3% |
| 2004 | Ecuador | Carchi | 10279 | 9452 | -8% | 5705 | -44% | 11958 | 16% | 10977 | 7% |
| 2004 | Ecuador | Manabí | 121833 | 31949 | -74% | 84398 | -31% | 57141 | -53% | 92893 | -24% |
| 2004 | Ecuador | Cañar | 13922 | 28552 | 105% | 20168 | 45% | 26616 | 91% | 22811 | 64% |
| 2004 | Ecuador | Bolívar | 19622 | 13689 | -30% | 23435 | 19% | 21127 | 8% | 18906 | -4% |
| 2004 | Ecuador | El Oro | 37884 | 19223 | -49% | 28253 | -25% | 21050 | -44% | 46291 | 22% |
| 2004 | Ecuador | Esmeraldas | 30872 | 30778 | 0% | 34746 | 13% | 23323 | -24% | 34875 | 13% |
| 2004 | Ecuador | Azuay | 35095 | 112305 | 220% | 37575 | 7% | 55639 | 59% | 40616 | 16% |
| 2004 | Ecuador | Zamora-Chinchipe | 7861 | 6250 | -20% | 8667 | 10% | 13067 | 66% | 6092 | -23% |
| 2004 | Ecuador | Guayas | 220824 | 94147 | -57% | 185734 | -16% | 140953 | -36% | 178872 | -19% |
| 2004 | Ecuador | Tungurahua | 31287 | 21654 | -31% | 29920 | -4% | 47911 | 53% | 21024 | -33% |
| 2004 | Ecuador | Chimborazo | 37550 | 37833 | 1% | 46022 | 23% | 48116 | 28% | 41431 | 10% |
| 2004 | Ecuador | Los Ríos | 55536 | 13542 | -76% | 44295 | -20% | 40442 | -27% | 60291 | 9% |
| 2004 | Ecuador | Sucumbíos | 11795 | 7661 | -35% | 10330 | -12% | 6001 | -49% | 18486 | 57% |
| 2004 | Ecuador | Cotopaxi | 30546 | 33223 | 9% | 40658 | 33% | 39379 | 29% | 29059 | -5% |
| 2004 | Ecuador | Imbabura | 26144 | 43380 | 66% | 20453 | -22% | 34779 | 33% | 29461 | 13% |
| 2012 | Ecuador | Zamora-Chinchipe | 10110 |  |  | 7333 | -27% | 9366 | -7% | 10398 | 3% |
| 2012 | Ecuador | Imbabura | 33600 |  |  | 37650 | 12% | 45172 | 34% | 25517 | -24% |
| 2012 | Ecuador | Guayas | 319622 |  |  | 363887 | 14% | 307155 | -4% | 297969 | -7% |
| 2012 | Ecuador | Los Ríos | 100407 |  |  | 78920 | -21% | 43384 | -57% | 71006 | -29% |
| 2012 | Ecuador | Tungurahua | 40244 |  |  | 40447 | 1% | 52516 | 30% | 34292 | -15% |
| 2012 | Ecuador | Bolívar | 25443 |  |  | 24110 | -5% | 28524 | 12% | 24142 | -5% |
| 2012 | Ecuador | Manabí | 169115 |  |  | 85477 | -49% | 85992 | -49% | 128372 | -24% |
| 2012 | Ecuador | Cañar | 20764 |  |  | 23026 | 11% | 29159 | 40% | 34640 | 67% |
| 2012 | Ecuador | Pichincha | 102290 |  |  | 212060 | 107% | 199246 | 95% | 160413 | 57% |
| 2012 | Ecuador | Chimborazo | 47598 |  |  | 66644 | 40% | 68979 | 45% | 65569 | 38% |
| 2012 | Ecuador | Galápagos | 339 |  |  | 1229 | 263% | 1207 | 256% | 1526 | 350% |
| 2012 | Ecuador | Pastaza | 6712 |  |  | 5359 | -20% | 9640 | 44% | 7850 | 17% |
| 2012 | Ecuador | Napo | 27621 |  |  | 20743 | -25% | 23487 | -15% | 25736 | -7% |
| 2012 | Ecuador | El Oro | 43583 |  |  | 23582 | -46% | 26957 | -38% | 41050 | -6% |
| 2012 | Ecuador | Carchi | 14473 |  |  | 12307 | -15% | 17768 | 23% | 24617 | 70% |
| 2012 | Ecuador | Morona Santiago | 15835 |  |  | 17704 | 12% | 21971 | 39% | 19496 | 23% |
| 2012 | Ecuador | Azuay | 47963 |  |  | 55485 | 16% | 75459 | 57% | 54659 | 14% |
| 2012 | Ecuador | Esmeraldas | 63067 |  |  | 47530 | -25% | 40306 | -36% | 69160 | 10% |
| 2012 | Ecuador | Cotopaxi | 48116 |  |  | 25252 | -48% | 56524 | 17% | 52016 | 8% |
| 2012 | Ecuador | Sucumbíos | 19887 |  |  | 10584 | -47% | 12591 | -37% | 16457 | -17% |
| 2012 | Ecuador | Loja | 41454 |  |  | 38912 | -6% | 42840 | 3% | 33358 | -20% |
| 2009 | Peru | San Martin | 0 | 231 | N/A | 352 | N/A | 287 | N/A | 301 | N/A |
| 2009 | Peru | Tumbes | 0 | 46 | N/A | 54 | N/A | 35 | N/A | 53 | N/A |
| 2009 | Peru | Piura | 795 | 549 | -31% | 598 | -25% | 520 | -35% | 672 | -16% |
| 2009 | Peru | Junín | 481 | 427 | -11% | 601 | 25% | 665 | 38% | 478 | -1% |
| 2009 | Peru | Ica | 0 | 248 | N/A | 101 | N/A | 95 | N/A | 129 | N/A |
| 2009 | Peru | Cajamarca | 810 | 497 | -39% | 949 | 17% | 911 | 12% | 544 | -33% |
| 2009 | Peru | Amazonas | 203 | 138 | -32% | 206 | 2% | 177 | -13% | 134 | -34% |
| 2009 | Peru | Cusco | 1041 | 359 | -66% | 797 | -23% | 685 | -34% | 514 | -51% |
| 2009 | Peru | La Libertad | 601 | 559 | -7% | 605 | 1% | 606 | 1% | 418 | -31% |
| 2009 | Peru | Pasco | 72 | 109 | 51% | 150 | 108% | 160 | 122% | 133 | 85% |
| 2009 | Peru | Ucayali | 0 | 187 | N/A | 329 | N/A | 186 | N/A | 209 | N/A |
| 2009 | Peru | Lima | 0 | 2307 | N/A | 409 | N/A | 675 | N/A | 1193 | N/A |
| 2009 | Peru | Ayacucho | 915 | 234 | -74% | 472 | -48% | 404 | -56% | 347 | -62% |
| 2009 | Peru | Huánuco | 1009 | 288 | -71% | 378 | -63% | 535 | -47% | 352 | -65% |
| 2009 | Peru | Apurímac | 459 | 144 | -69% | 215 | -53% | 251 | -45% | 225 | -51% |
| 2009 | Peru | Madre de Dios | 0 | 54 | N/A | 16 | N/A | 18 | N/A | 40 | N/A |
| 2009 | Peru | Puno | 330 | 518 | 57% | 238 | -28% | 497 | 50% | 811 | 146% |
| 2009 | Peru | Callao | 0 |  | N/A |  | N/A |  | N/A | 112 | N/A |
| 2009 | Peru | Tacna | 0 | 90 | N/A | 6 | N/A | 10 | N/A | 70 | N/A |
| 2009 | Peru | Huancavelica | 572 | 132 | -77% | 472 | -18% | 479 | -16% | 328 | -43% |
| 2009 | Peru | Arequipa | 0 | 278 | N/A | 113 | N/A | 146 | N/A | 209 | N/A |
| 2009 | Peru | Loreto | 429 | 453 | 6% | 834 | 94% | 481 | 12% | 638 | 49% |
| 2009 | Peru | Moquegua | 0 | 33 | N/A | 0 | N/A | 10 | N/A | 38 | N/A |
| 2009 | Peru | Lambayeque | 0 | 371 | N/A | 181 | N/A | 244 | N/A | 249 | N/A |
| 2009 | Peru | Ancash | 800 | 266 | -67% | 444 | -45% | 443 | -45% | 319 | -60% |
| 2010 | Peru | Apurímac | 307 | 136 | -56% | 344 | 12% | 329 | 7% | 256 | -17% |
| 2010 | Peru | San Martin | 0 | 236 | N/A | 429 | N/A | 285 | N/A | 236 | N/A |
| 2010 | Peru | Loreto | 256 | 437 | 71% | 759 | 196% | 590 | 130% | 626 | 145% |
| 2010 | Peru | Huánuco | 870 | 307 | -65% | 401 | -54% | 523 | -40% | 394 | -55% |
| 2010 | Peru | Lambayeque | 0 | 304 | N/A | 263 | N/A | 277 | N/A | 356 | N/A |
| 2010 | Peru | Lima | 0 | 2573 | N/A | 740 | N/A | 906 | N/A | 1692 | N/A |
| 2010 | Peru | Callao | 0 |  | N/A |  | N/A |  | N/A | 160 | N/A |
| 2010 | Peru | Moquegua | 0 | 51 | N/A | 10 | N/A | 13 | N/A | 27 | N/A |
| 2010 | Peru | La Libertad | 780 | 492 | -37% | 548 | -30% | 562 | -28% | 427 | -45% |
| 2010 | Peru | Ica | 0 | 283 | N/A | 102 | N/A | 98 | N/A | 126 | N/A |
| 2010 | Peru | Tacna | 0 | 77 | N/A | 9 | N/A | 15 | N/A | 47 | N/A |
| 2010 | Peru | Piura | 1141 | 668 | -41% | 529 | -54% | 568 | -50% | 536 | -53% |
| 2010 | Peru | Tumbes | 0 | 53 | N/A | 50 | N/A | 32 | N/A | 59 | N/A |
| 2010 | Peru | Amazonas | 306 | 177 | -42% | 183 | -40% | 147 | -52% | 147 | -52% |
| 2010 | Peru | Junín | 613 | 412 | -33% | 550 | -10% | 561 | -8% | 510 | -17% |
| 2010 | Peru | Ancash | 725 | 294 | -59% | 362 | -50% | 516 | -29% | 288 | -60% |
| 2010 | Peru | Ucayali | 0 | 181 | N/A | 447 | N/A | 238 | N/A | 206 | N/A |
| 2010 | Peru | Puno | 510 | 619 | 21% | 391 | -23% | 510 | 0% | 754 | 48% |
| 2010 | Peru | Huancavelica | 518 | 109 | -79% | 795 | 53% | 593 | 14% | 427 | -18% |
| 2010 | Peru | Arequipa | 0 | 359 | N/A | 198 | N/A | 149 | N/A | 193 | N/A |
| 2010 | Peru | Madre de Dios | 0 | 54 | N/A | 31 | N/A | 18 | N/A | 40 | N/A |
| 2010 | Peru | Cusco | 950 | 342 | -64% | 758 | -20% | 674 | -29% | 641 | -33% |
| 2010 | Peru | Ayacucho | 917 | 234 | -74% | 347 | -62% | 424 | -54% | 373 | -59% |
| 2010 | Peru | Pasco | 116 | 115 | -1% | 83 | -29% | 122 | 5% | 140 | 20% |
| 2010 | Peru | Cajamarca | 1114 | 608 | -45% | 794 | -29% | 972 | -13% | 461 | -59% |
| 2011 | Peru | Tumbes | 0 | 73 | N/A | 52 | N/A | 33 | N/A | 64 | N/A |
| 2011 | Peru | San Martin | 0 | 283 | N/A | 545 | N/A | 280 | N/A | 427 | N/A |
| 2011 | Peru | Madre de Dios | 0 | 71 | N/A | 37 | N/A | 22 | N/A | 58 | N/A |
| 2011 | Peru | Lima | 0 | 2463 | N/A | 950 | N/A | 859 | N/A | 1366 | N/A |
| 2011 | Peru | Huancavelica | 889 | 151 | -83% | 676 | -24% | 714 | -20% | 517 | -42% |
| 2011 | Peru | Ancash | 803 | 451 | -44% | 402 | -50% | 446 | -44% | 302 | -62% |
| 2011 | Peru | Amazonas | 233 | 207 | -11% | 284 | 22% | 270 | 16% | 157 | -33% |
| 2011 | Peru | Cajamarca | 1369 | 540 | -61% | 977 | -29% | 1022 | -25% | 628 | -54% |
| 2011 | Peru | Piura | 1280 | 679 | -47% | 514 | -60% | 675 | -47% | 726 | -43% |
| 2011 | Peru | Pasco | 107 | 100 | -7% | 121 | 13% | 130 | 22% | 162 | 51% |
| 2011 | Peru | Callao | 0 |  | N/A |  | N/A |  | N/A | 107 | N/A |
| 2011 | Peru | La Libertad | 638 | 620 | -3% | 626 | -2% | 712 | 12% | 344 | -46% |
| 2011 | Peru | Apurímac | 552 | 154 | -72% | 347 | -37% | 347 | -37% | 275 | -50% |
| 2011 | Peru | Huánuco | 930 | 372 | -60% | 557 | -40% | 601 | -35% | 421 | -55% |
| 2011 | Peru | Tacna | 0 | 103 | N/A | 9 | N/A | 20 | N/A | 74 | N/A |
| 2011 | Peru | Ica | 0 | 328 | N/A | 73 | N/A | 89 | N/A | 222 | N/A |
| 2011 | Peru | Ucayali | 0 | 206 | N/A | 277 | N/A | 222 | N/A | 175 | N/A |
| 2011 | Peru | Puno | 470 | 696 | 48% | 374 | -21% | 489 | 4% | 1052 | 124% |
| 2011 | Peru | Loreto | 197 | 489 | 148% | 1030 | 423% | 721 | 266% | 528 | 168% |
| 2011 | Peru | Moquegua | 0 | 38 | N/A | 13 | N/A | 11 | N/A | 43 | N/A |
| 2011 | Peru | Lambayeque | 0 | 410 | N/A | 144 | N/A | 334 | N/A | 395 | N/A |
| 2011 | Peru | Ayacucho | 1377 | 318 | -77% | 338 | -75% | 470 | -66% | 406 | -70% |
| 2011 | Peru | Junín | 442 | 459 | 4% | 835 | 89% | 748 | 69% | 595 | 35% |
| 2011 | Peru | Cusco | 667 | 371 | -44% | 675 | 1% | 643 | -4% | 754 | 13% |
| 2011 | Peru | Arequipa | 0 | 372 | N/A | 100 | N/A | 95 | N/A | 158 | N/A |
| 2012 | Peru | Lambayeque | 0 | 956 | N/A | 968 | N/A | 609 | N/A | 555 | N/A |
| 2012 | Peru | Ayacucho | 1478 | 644 | -56% | 882 | -40% | 900 | -39% | 760 | -49% |
| 2012 | Peru | La Libertad | 1496 | 1034 | -31% | 1049 | -30% | 1572 | 5% | 622 | -58% |
| 2012 | Peru | Tacna | 0 | 220 | N/A | 15 | N/A | 26 | N/A | 154 | N/A |
| 2012 | Peru | Apurímac | 931 | 295 | -68% | 551 | -41% | 634 | -32% | 492 | -47% |
| 2012 | Peru | Huancavelica | 1081 | 353 | -67% | 1389 | 28% | 1408 | 30% | 895 | -17% |
| 2012 | Peru | Pasco | 334 | 289 | -13% | 437 | 31% | 377 | 13% | 353 | 6% |
| 2012 | Peru | Cajamarca | 3249 | 1313 | -60% | 1207 | -63% | 2348 | -28% | 1436 | -56% |
| 2012 | Peru | Moquegua | 0 | 78 | N/A | 38 | N/A | 23 | N/A | 58 | N/A |
| 2012 | Peru | Amazonas | 1032 | 418 | -60% | 678 | -34% | 534 | -48% | 341 | -67% |
| 2012 | Peru | Junín | 632 | 943 | 49% | 999 | 58% | 1160 | 84% | 1348 | 113% |
| 2012 | Peru | Callao | 0 |  | N/A |  | N/A |  | N/A | 285 | N/A |
| 2012 | Peru | Ica | 0 | 645 | N/A | 185 | N/A | 185 | N/A | 371 | N/A |
| 2012 | Peru | Piura | 2650 | 1346 | -49% | 1529 | -42% | 1728 | -35% | 1038 | -61% |
| 2012 | Peru | Huánuco | 1256 | 643 | -49% | 1184 | -6% | 1126 | -10% | 1163 | -7% |
| 2012 | Peru | Arequipa | 0 | 708 | N/A | 240 | N/A | 384 | N/A | 377 | N/A |
| 2012 | Peru | Tumbes | 0 | 148 | N/A | 94 | N/A | 60 | N/A | 132 | N/A |
| 2012 | Peru | Cusco | 1402 | 817 | -42% | 1316 | -6% | 1182 | -16% | 1547 | 10% |
| 2012 | Peru | Ancash | 1282 | 843 | -34% | 1023 | -20% | 1053 | -18% | 764 | -40% |
| 2012 | Peru | Puno | 727 | 1416 | 95% | 1260 | 73% | 1423 | 96% | 2229 | 207% |
| 2012 | Peru | San Martin | 0 | 612 | N/A | 892 | N/A | 472 | N/A | 686 | N/A |
| 2012 | Peru | Loreto | 3485 | 971 | -72% | 1896 | -46% | 1503 | -57% | 1354 | -61% |
| 2012 | Peru | Ucayali | 0 | 433 | N/A | 347 | N/A | 355 | N/A | 355 | N/A |
| 2012 | Peru | Lima | 0 | 5794 | N/A | 2775 | N/A | 1914 | N/A | 3603 | N/A |
| 2012 | Peru | Madre de Dios | 0 | 117 | N/A | 81 | N/A | 59 | N/A | 118 | N/A |

Table 16. Simple prediction model with the actual number of CCT enrollees per subregion, the number of enrollees expected in that subregion with simple predictive targeting for each health outcome, and the percent deviation between the actual and expected targeting.

|  |  |  | Actual | DPT | | Underweight | | Stunting | | Child deaths | |
| --- | --- | --- | --- | --- | --- | --- | --- | --- | --- | --- | --- |
| Year | Country | Region | # | # | % ∆ | # | % ∆ | # | % ∆ | # | % ∆ |
| 2012 | Bolivia | La Paz | 19588 | 15993 | -18% | 13117 | -33% | 13980 | -29% | 14656 | -25% |
| 2012 | Bolivia | Santa Cruz | 8392 | 18626 | 122% | 11331 | 35% | 13848 | 65% | 15915 | 90% |
| 2012 | Bolivia | Tarija | 4029 | 3171 | -21% | 1310 | -67% | 1962 | -51% | 2499 | -38% |
| 2012 | Bolivia | Cochabamba | 10416 | 11395 | 9% | 11563 | 11% | 11465 | 10% | 11376 | 9% |
| 2012 | Bolivia | Oruro | 3710 | 3161 | -15% | 4643 | 25% | 4080 | 10% | 3605 | -3% |
| 2012 | Bolivia | Pando | 185 | 882 | 377% | 1152 | 522% | 1018 | 450% | 897 | 385% |
| 2012 | Bolivia | Chuquisaca | 6295 | 3435 | -45% | 6910 | 10% | 5996 | -5% | 5317 | -16% |
| 2012 | Bolivia | Beni | 3740 | 3091 | -17% | 5432 | 45% | 4470 | 20% | 3641 | -3% |
| 2012 | Bolivia | Potosí | 8371 | 4973 | -41% | 9268 | 11% | 7907 | -6% | 6819 | -19% |
| 2005 | Colombia | Antioquia | 66760 |  |  |  |  |  |  |  |  |
| 2005 | Colombia | Guainía | 446 |  |  |  |  |  |  |  |  |
| 2005 | Colombia | Casanare | 9312 |  |  |  |  |  |  |  |  |
| 2005 | Colombia | Santander | 25528 |  |  |  |  |  |  |  |  |
| 2005 | Colombia | Arauca | 9609 |  |  |  |  |  |  |  |  |
| 2005 | Colombia | Guaviare | 2899 |  |  |  |  |  |  |  |  |
| 2005 | Colombia | Cauca | 30521 |  |  |  |  |  |  |  |  |
| 2005 | Colombia | Meta | 13177 |  |  |  |  |  |  |  |  |
| 2005 | Colombia | Bolívar | 43019 |  |  |  |  |  |  |  |  |
| 2005 | Colombia | Huila | 29784 |  |  |  |  |  |  |  |  |
| 2005 | Colombia | Córdoba | 53564 |  |  |  |  |  |  |  |  |
| 2005 | Colombia | Putumayo | 10251 |  |  |  |  |  |  |  |  |
| 2005 | Colombia | Nariño | 37832 |  |  |  |  |  |  |  |  |
| 2005 | Colombia | Chocó | 16315 |  |  |  |  |  |  |  |  |
| 2005 | Colombia | Caquetá | 14387 |  |  |  |  |  |  |  |  |
| 2005 | Colombia | Caldas | 8829 |  |  |  |  |  |  |  |  |
| 2005 | Colombia | San Andrés y Providencia | 1302 |  |  |  |  |  |  |  |  |
| 2005 | Colombia | Vichada | 902 |  |  |  |  |  |  |  |  |
| 2005 | Colombia | Bogotá | 7640 |  |  |  |  |  |  |  |  |
| 2005 | Colombia | Sucre | 37458 |  |  |  |  |  |  |  |  |
| 2005 | Colombia | Atlántico | 18505 |  |  |  |  |  |  |  |  |
| 2005 | Colombia | Boyacá | 25398 |  |  |  |  |  |  |  |  |
| 2005 | Colombia | Vaupés | 292 |  |  |  |  |  |  |  |  |
| 2005 | Colombia | Magdalena | 32410 |  |  |  |  |  |  |  |  |
| 2005 | Colombia | Valle del Cauca | 24326 |  |  |  |  |  |  |  |  |
| 2005 | Colombia | Risaralda | 5783 |  |  |  |  |  |  |  |  |
| 2005 | Colombia | Quindío | 10614 |  |  |  |  |  |  |  |  |
| 2005 | Colombia | Amazonas | 665 |  |  |  |  |  |  |  |  |
| 2005 | Colombia | La Guajira | 11953 |  |  |  |  |  |  |  |  |
| 2005 | Colombia | Cundinamarca | 33148 |  |  |  |  |  |  |  |  |
| 2005 | Colombia | Tolima | 32703 |  |  |  |  |  |  |  |  |
| 2005 | Colombia | Cesar | 28974 |  |  |  |  |  |  |  |  |
| 2005 | Colombia | Norte de Santander | 21009 |  |  |  |  |  |  |  |  |
| 2010 | Colombia | Nariño | 128705 | 116567 | -9% | 138597 | 8% | 132802 | 3% | 119064 | -7% |
| 2010 | Colombia | Bolívar | 182870 | 151818 | -17% | 173322 | -5% | 153679 | -16% | 142441 | -22% |
| 2010 | Colombia | Córdoba | 170938 | 113807 | -33% | 145995 | -15% | 162154 | -5% | 138259 | -19% |
| 2010 | Colombia | Vichada | 2346 | 5617 | 139% | 7671 | 227% | 9293 | 296% | 7676 | 227% |
| 2010 | Colombia | Cesar | 81878 | 74689 | -9% | 79686 | -3% | 71524 | -13% | 69471 | -15% |
| 2010 | Colombia | Norte de Santander | 83932 | 73542 | -12% | 72710 | -13% | 72818 | -13% | 73354 | -13% |
| 2010 | Colombia | Valle del Cauca | 142910 | 163051 | 14% | 121938 | -15% | 141621 | -1% | 165882 | 16% |
| 2010 | Colombia | Amazonas | 3998 | 6003 | 50% | 8133 | 103% | 9989 | 150% | 8286 | 107% |
| 2010 | Colombia | Caldas | 39463 | 41555 | 5% | 36052 | -9% | 38985 | -1% | 42186 | 7% |
| 2010 | Colombia | Magdalena | 119616 | 101623 | -15% | 117594 | -2% | 107256 | -10% | 98249 | -18% |
| 2010 | Colombia | Cauca | 120416 | 93174 | -23% | 114928 | -5% | 113562 | -6% | 99320 | -18% |
| 2010 | Colombia | Guainía | 857 | 2948 | 244% | 3941 | 360% | 4805 | 461% | 4011 | 368% |
| 2010 | Colombia | Arauca | 25424 | 15654 | -38% | 12599 | -50% | 14505 | -43% | 16239 | -36% |
| 2010 | Colombia | Quindío | 26690 | 19785 | -26% | 13956 | -48% | 17190 | -36% | 20560 | -23% |
| 2010 | Colombia | San Andrés y Providencia | 1663 | 4357 | 162% | 4718 | 184% | 4246 | 155% | 4079 | 145% |
| 2010 | Colombia | Casanare | 32443 | 19070 | -41% | 17597 | -46% | 17996 | -45% | 18913 | -42% |
| 2010 | Colombia | Meta | 49266 | 50916 | 3% | 48359 | -2% | 47351 | -4% | 49206 | 0% |
| 2010 | Colombia | Antioquia | 246401 | 272049 | 10% | 244261 | -1% | 253581 | 3% | 270597 | 10% |
| 2010 | Colombia | Guaviare | 8376 | 9073 | 8% | 9524 | 14% | 8728 | 4% | 8552 | 2% |
| 2010 | Colombia | Atlántico | 140564 | 101548 | -28% | 79588 | -43% | 89000 | -37% | 102130 | -27% |
| 2010 | Colombia | Cundinamarca | 100967 | 138481 | 37% | 134910 | 34% | 127108 | 26% | 130692 | 29% |
| 2010 | Colombia | Sucre | 94085 | 60006 | -36% | 70407 | -25% | 66172 | -30% | 59919 | -36% |
| 2010 | Colombia | La Guajira | 48251 | 81615 | 69% | 99466 | 106% | 97330 | 102% | 85801 | 78% |
| 2010 | Colombia | Risaralda | 39188 | 36518 | -7% | 30403 | -22% | 34760 | -11% | 38147 | -3% |
| 2010 | Colombia | Putumayo | 32021 | 27856 | -13% | 33568 | 5% | 32828 | 3% | 29148 | -9% |
| 2010 | Colombia | Huila | 93076 | 79206 | -15% | 88203 | -5% | 79501 | -15% | 74874 | -20% |
| 2010 | Colombia | Santander | 98039 | 106695 | 9% | 110030 | 12% | 103033 | 5% | 101904 | 4% |
| 2010 | Colombia | Chocó | 32053 | 37515 | 17% | 49832 | 55% | 62470 | 95% | 52324 | 63% |
| 2010 | Colombia | Caquetá | 44468 | 37164 | -16% | 40824 | -8% | 36894 | -17% | 35072 | -21% |
| 2010 | Colombia | Bogotá | 127577 | 278952 | 119% | 199442 | 56% | 207824 | 63% | 259351 | 103% |
| 2010 | Colombia | Vaupés | 490 | 3736 | 662% | 4748 | 869% | 5350 | 992% | 4584 | 835% |
| 2010 | Colombia | Boyacá | 71285 | 83777 | 18% | 94532 | 33% | 85458 | 20% | 79721 | 12% |
| 2010 | Colombia | Tolima | 96477 | 78365 | -19% | 79197 | -18% | 76920 | -20% | 76721 | -20% |
| 2015 | Colombia | Nariño | 155777 | 121612 | -22% | 151381 | -3% | 150114 | -4% | 130378 | -16% |
| 2015 | Colombia | Cauca | 138329 | 94873 | -31% | 117296 | -15% | 119137 | -14% | 103849 | -25% |
| 2015 | Colombia | Cundinamarca | 89980 | 148547 | 65% | 146267 | 63% | 137730 | 53% | 140547 | 56% |
| 2015 | Colombia | Bogotá | 68085 | 285102 | 319% | 202295 | 197% | 211774 | 211% | 265773 | 290% |
| 2015 | Colombia | Sucre | 92576 | 63274 | -32% | 75026 | -19% | 69946 | -24% | 62847 | -32% |
| 2015 | Colombia | Vichada | 2320 | 6435 | 177% | 6468 | 179% | 5905 | 155% | 5968 | 157% |
| 2015 | Colombia | Vaupés | 1092 | 2763 | 153% | 3974 | 264% | 6091 | 458% | 4960 | 354% |
| 2015 | Colombia | Meta | 61755 | 47659 | -23% | 41012 | -34% | 44429 | -28% | 48362 | -22% |
| 2015 | Colombia | Córdoba | 193620 | 125876 | -35% | 154264 | -20% | 159056 | -18% | 139328 | -28% |
| 2015 | Colombia | Guainía | 2219 | 2438 | 10% | 3442 | 55% | 5202 | 134% | 4265 | 92% |
| 2015 | Colombia | Guaviare | 8515 | 8163 | -4% | 7669 | -10% | 7650 | -10% | 7983 | -6% |
| 2015 | Colombia | Boyacá | 62514 | 80905 | 29% | 93684 | 50% | 85628 | 37% | 78291 | 25% |
| 2015 | Colombia | Antioquia | 296873 | 285837 | -4% | 255124 | -14% | 261234 | -12% | 280870 | -5% |
| 2015 | Colombia | Atlántico | 116414 | 99117 | -15% | 71738 | -38% | 80024 | -31% | 97099 | -17% |
| 2015 | Colombia | Cesar | 94867 | 73987 | -22% | 78120 | -18% | 71578 | -25% | 69799 | -26% |
| 2015 | Colombia | Norte de Santander | 99377 | 74862 | -25% | 72109 | -27% | 69582 | -30% | 71807 | -28% |
| 2015 | Colombia | Amazonas | 5111 | 5942 | 16% | 7805 | 53% | 9867 | 93% | 8308 | 63% |
| 2015 | Colombia | San Andrés y Providencia | 2493 | 3110 | 25% | 2510 | 1% | 2775 | 11% | 3137 | 26% |
| 2015 | Colombia | Arauca | 24548 | 20922 | -15% | 22770 | -7% | 23047 | -6% | 21768 | -11% |
| 2015 | Colombia | Casanare | 33399 | 19608 | -41% | 17894 | -46% | 18647 | -44% | 19681 | -41% |
| 2015 | Colombia | Caquetá | 45129 | 40106 | -11% | 47038 | 4% | 43860 | -3% | 39700 | -12% |
| 2015 | Colombia | Santander | 98584 | 108539 | 10% | 112780 | 14% | 104375 | 6% | 102806 | 4% |
| 2015 | Colombia | Magdalena | 122257 | 95032 | -22% | 106284 | -13% | 101206 | -17% | 94441 | -23% |
| 2015 | Colombia | Risaralda | 39034 | 37481 | -4% | 31208 | -20% | 34462 | -12% | 38169 | -2% |
| 2015 | Colombia | Huila | 96937 | 80636 | -17% | 91299 | -6% | 85540 | -12% | 79272 | -18% |
| 2015 | Colombia | La Guajira | 61519 | 90819 | 48% | 109566 | 78% | 108708 | 77% | 96288 | 57% |
| 2015 | Colombia | Valle del Cauca | 139072 | 183308 | 32% | 145829 | 5% | 149615 | 8% | 174131 | 25% |
| 2015 | Colombia | Tolima | 97856 | 79535 | -19% | 85212 | -13% | 85505 | -13% | 81660 | -17% |
| 2015 | Colombia | Quindío | 19068 | 22264 | 17% | 16971 | -11% | 18012 | -6% | 21397 | 12% |
| 2015 | Colombia | Caldas | 41683 | 50759 | 22% | 51855 | 24% | 48341 | 16% | 48138 | 15% |
| 2015 | Colombia | Bolívar | 169218 | 135788 | -20% | 149909 | -11% | 146857 | -13% | 137865 | -19% |
| 2015 | Colombia | Putumayo | 37462 | 28881 | -23% | 33068 | -12% | 30379 | -19% | 27982 | -25% |
| 2015 | Colombia | Chocó | 42271 | 35773 | -15% | 48086 | 14% | 63680 | 51% | 53081 | 26% |
| 2004 | Ecuador | Morona Santiago | 8177 | 11406 | 39% | 10559 | 29% | 10907 | 33% | 11046 | 35% |
| 2004 | Ecuador | Pastaza | 4213 | 5957 | 41% | 6378 | 51% | 5916 | 40% | 5700 | 35% |
| 2004 | Ecuador | Napo | 14557 | 17633 | 21% | 20929 | 44% | 18889 | 30% | 17998 | 24% |
| 2004 | Ecuador | Galápagos | 247 | 1194 | 383% | 872 | 253% | 994 | 302% | 1042 | 322% |
| 2004 | Ecuador | Pichincha | 89030 | 147351 | 66% | 121845 | 37% | 134021 | 51% | 139082 | 56% |
| 2004 | Ecuador | Loja | 32411 | 29876 | -8% | 33204 | 2% | 31260 | -4% | 30419 | -6% |
| 2004 | Ecuador | Carchi | 10279 | 9723 | -5% | 9431 | -8% | 9489 | -8% | 9505 | -8% |
| 2004 | Ecuador | Manabí | 121833 | 87023 | -29% | 94683 | -22% | 90811 | -25% | 89182 | -27% |
| 2004 | Ecuador | Cañar | 13922 | 15904 | 14% | 16893 | 21% | 16086 | 16% | 15719 | 13% |
| 2004 | Ecuador | Bolívar | 19622 | 11674 | -41% | 23284 | 19% | 20572 | 5% | 19709 | 0% |
| 2004 | Ecuador | El Oro | 37884 | 34509 | -9% | 31382 | -17% | 32849 | -13% | 33457 | -12% |
| 2004 | Ecuador | Esmeraldas | 30872 | 37577 | 22% | 42576 | 38% | 39078 | 27% | 37521 | 22% |
| 2004 | Ecuador | Azuay | 35095 | 43416 | 24% | 40126 | 14% | 39747 | 13% | 39408 | 12% |
| 2004 | Ecuador | Zamora-Chinchipe | 7861 | 7192 | -9% | 6813 | -13% | 6802 | -13% | 6779 | -14% |
| 2004 | Ecuador | Guayas | 220824 | 207781 | -6% | 194496 | -12% | 205087 | -7% | 209885 | -5% |
| 2004 | Ecuador | Tungurahua | 31287 | 25901 | -17% | 23642 | -24% | 25303 | -19% | 26048 | -17% |
| 2004 | Ecuador | Chimborazo | 37550 | 30807 | -18% | 40116 | 7% | 35872 | -4% | 34127 | -9% |
| 2004 | Ecuador | Los Ríos | 55536 | 51195 | -8% | 55585 | 0% | 51958 | -6% | 50308 | -9% |
| 2004 | Ecuador | Sucumbíos | 11795 | 10893 | -8% | 11870 | 1% | 11485 | -3% | 11333 | -4% |
| 2004 | Ecuador | Cotopaxi | 30546 | 28630 | -6% | 32480 | 6% | 29577 | -3% | 28271 | -7% |
| 2004 | Ecuador | Imbabura | 26144 | 24043 | -8% | 22519 | -14% | 22981 | -12% | 23148 | -11% |
| 2012 | Ecuador | Zamora-Chinchipe | 10110 | 10272 | 2% | 11398 | 13% | 10826 | 7% | 10587 | 5% |
| 2012 | Ecuador | Imbabura | 33600 | 31994 | -5% | 33461 | 0% | 33443 | 0% | 33498 | 0% |
| 2012 | Ecuador | Guayas | 319622 | 306552 | -4% | 287818 | -10% | 300566 | -6% | 306156 | -4% |
| 2012 | Ecuador | Los Ríos | 100407 | 70105 | -30% | 77028 | -23% | 74515 | -26% | 73553 | -27% |
| 2012 | Ecuador | Tungurahua | 40244 | 36049 | -10% | 35530 | -12% | 36480 | -9% | 36934 | -8% |
| 2012 | Ecuador | Bolívar | 25443 | 18580 | -27% | 26981 | 6% | 23742 | -7% | 22480 | -12% |
| 2012 | Ecuador | Manabí | 169115 | 113609 | -33% | 118185 | -30% | 115926 | -31% | 114990 | -32% |
| 2012 | Ecuador | Cañar | 20764 | 21197 | 2% | 24219 | 17% | 22578 | 9% | 21886 | 5% |
| 2012 | Ecuador | Pichincha | 102290 | 220305 | 115% | 182398 | 78% | 198653 | 94% | 205172 | 101% |
| 2012 | Ecuador | Chimborazo | 47598 | 41562 | -13% | 46408 | -2% | 42616 | -10% | 40922 | -14% |
| 2012 | Ecuador | Galápagos | 339 | 1949 | 475% | 1642 | 384% | 1805 | 432% | 1874 | 453% |
| 2012 | Ecuador | Pastaza | 6712 | 9484 | 41% | 10570 | 57% | 9629 | 43% | 9204 | 37% |
| 2012 | Ecuador | Napo | 27621 | 29836 | 8% | 34855 | 26% | 31223 | 13% | 29616 | 7% |
| 2012 | Ecuador | El Oro | 43583 | 45465 | 4% | 40710 | -7% | 43282 | -1% | 44368 | 2% |
| 2012 | Ecuador | Carchi | 14473 | 12363 | -15% | 12926 | -11% | 12795 | -12% | 12753 | -12% |
| 2012 | Ecuador | Morona Santiago | 15835 | 19632 | 24% | 21619 | 37% | 19550 | 23% | 18596 | 17% |
| 2012 | Ecuador | Azuay | 47963 | 55303 | 15% | 55691 | 16% | 56449 | 18% | 56846 | 19% |
| 2012 | Ecuador | Esmeraldas | 63067 | 57095 | -9% | 62804 | 0% | 59172 | -6% | 57596 | -9% |
| 2012 | Ecuador | Cotopaxi | 48116 | 38365 | -20% | 43663 | -9% | 41756 | -13% | 41029 | -15% |
| 2012 | Ecuador | Sucumbíos | 19887 | 18254 | -8% | 20338 | 2% | 18830 | -5% | 18163 | -9% |
| 2012 | Ecuador | Loja | 41454 | 40273 | -3% | 50002 | 21% | 44406 | 7% | 42018 | 1% |
| 2009 | Peru | San Martin | 0 | 238 | N/A | 344 | N/A | 354 | N/A | 295 | N/A |
| 2009 | Peru | Tumbes | 0 | 59 | N/A | 33 | N/A | 34 | N/A | 48 | N/A |
| 2009 | Peru | Piura | 795 | 549 | -31% | 563 | -29% | 597 | -25% | 587 | -26% |
| 2009 | Peru | Junín | 481 | 415 | -14% | 391 | -19% | 418 | -13% | 429 | -11% |
| 2009 | Peru | Ica | 0 | 209 | N/A | 87 | N/A | 76 | N/A | 144 | N/A |
| 2009 | Peru | Cajamarca | 810 | 478 | -41% | 938 | 16% | 947 | 17% | 691 | -15% |
| 2009 | Peru | Amazonas | 203 | 136 | -33% | 221 | 9% | 222 | 10% | 175 | -14% |
| 2009 | Peru | Cusco | 1041 | 378 | -64% | 577 | -45% | 600 | -42% | 488 | -53% |
| 2009 | Peru | La Libertad | 601 | 501 | -17% | 549 | -9% | 601 | 0% | 570 | -5% |
| 2009 | Peru | Pasco | 72 | 97 | 35% | 84 | 17% | 88 | 23% | 95 | 32% |
| 2009 | Peru | Ucayali | 0 | 144 | N/A | 184 | N/A | 191 | N/A | 168 | N/A |
| 2009 | Peru | Lima | 0 | 2321 | N/A | 815 | N/A | 596 | N/A | 1446 | N/A |
| 2009 | Peru | Ayacucho | 915 | 225 | -75% | 383 | -58% | 393 | -57% | 305 | -67% |
| 2009 | Peru | Huánuco | 1009 | 277 | -73% | 518 | -49% | 533 | -47% | 398 | -61% |
| 2009 | Peru | Apurímac | 459 | 149 | -67% | 305 | -33% | 311 | -32% | 224 | -51% |
| 2009 | Peru | Madre de Dios | 0 | 38 | N/A | 30 | N/A | 31 | N/A | 35 | N/A |
| 2009 | Peru | Puno | 330 | 436 | 32% | 587 | 78% | 629 | 91% | 542 | 64% |
| 2009 | Peru | Callao | 0 | 233 | N/A | 51 | N/A | 14 | N/A | 117 | N/A |
| 2009 | Peru | Tacna | 0 | 86 | N/A | 24 | N/A | 14 | N/A | 49 | N/A |
| 2009 | Peru | Huancavelica | 572 | 189 | -67% | 404 | -29% | 410 | -28% | 291 | -49% |
| 2009 | Peru | Arequipa | 0 | 301 | N/A | 145 | N/A | 141 | N/A | 228 | N/A |
| 2009 | Peru | Loreto | 429 | 349 | -19% | 623 | 45% | 640 | 49% | 488 | 14% |
| 2009 | Peru | Moquegua | 0 | 40 | N/A | 25 | N/A | 27 | N/A | 35 | N/A |
| 2009 | Peru | Lambayeque | 0 | 335 | N/A | 236 | N/A | 250 | N/A | 303 | N/A |
| 2009 | Peru | Ancash | 800 | 332 | -59% | 401 | -50% | 401 | -50% | 363 | -55% |
| 2010 | Peru | Apurímac | 307 | 161 | -48% | 329 | 7% | 329 | 7% | 236 | -23% |
| 2010 | Peru | San Martin | 0 | 256 | N/A | 368 | N/A | 383 | N/A | 320 | N/A |
| 2010 | Peru | Loreto | 256 | 376 | 47% | 647 | 153% | 625 | 144% | 477 | 86% |
| 2010 | Peru | Huánuco | 870 | 298 | -66% | 560 | -36% | 556 | -36% | 411 | -53% |
| 2010 | Peru | Lambayeque | 0 | 357 | N/A | 251 | N/A | 264 | N/A | 321 | N/A |
| 2010 | Peru | Lima | 0 | 2494 | N/A | 855 | N/A | 673 | N/A | 1594 | N/A |
| 2010 | Peru | Callao | 0 | 251 | N/A | 54 | N/A | 16 | N/A | 128 | N/A |
| 2010 | Peru | Moquegua | 0 | 43 | N/A | 27 | N/A | 28 | N/A | 37 | N/A |
| 2010 | Peru | La Libertad | 780 | 539 | -31% | 546 | -30% | 597 | -23% | 589 | -24% |
| 2010 | Peru | Ica | 0 | 221 | N/A | 120 | N/A | 129 | N/A | 184 | N/A |
| 2010 | Peru | Tacna | 0 | 93 | N/A | 26 | N/A | 15 | N/A | 53 | N/A |
| 2010 | Peru | Piura | 1141 | 584 | -49% | 636 | -44% | 704 | -38% | 670 | -41% |
| 2010 | Peru | Tumbes | 0 | 65 | N/A | 26 | N/A | 24 | N/A | 45 | N/A |
| 2010 | Peru | Amazonas | 306 | 145 | -53% | 260 | -15% | 259 | -16% | 195 | -36% |
| 2010 | Peru | Junín | 613 | 446 | -27% | 384 | -37% | 406 | -34% | 438 | -28% |
| 2010 | Peru | Ancash | 725 | 353 | -51% | 451 | -38% | 465 | -36% | 409 | -44% |
| 2010 | Peru | Ucayali | 0 | 152 | N/A | 179 | N/A | 194 | N/A | 178 | N/A |
| 2010 | Peru | Puno | 510 | 466 | -9% | 632 | 24% | 659 | 29% | 565 | 11% |
| 2010 | Peru | Huancavelica | 518 | 205 | -60% | 402 | -22% | 399 | -23% | 290 | -44% |
| 2010 | Peru | Arequipa | 0 | 323 | N/A | 153 | N/A | 156 | N/A | 250 | N/A |
| 2010 | Peru | Madre de Dios | 0 | 41 | N/A | 32 | N/A | 34 | N/A | 39 | N/A |
| 2010 | Peru | Cusco | 950 | 404 | -57% | 616 | -35% | 635 | -33% | 516 | -46% |
| 2010 | Peru | Ayacucho | 917 | 244 | -73% | 400 | -56% | 385 | -58% | 300 | -67% |
| 2010 | Peru | Pasco | 116 | 103 | -11% | 97 | -16% | 102 | -12% | 105 | -10% |
| 2010 | Peru | Cajamarca | 1114 | 505 | -55% | 1074 | -4% | 1087 | -2% | 771 | -31% |
| 2011 | Peru | Tumbes | 0 | 70 | N/A | 35 | N/A | 35 | N/A | 56 | N/A |
| 2011 | Peru | San Martin | 0 | 279 | N/A | 388 | N/A | 428 | N/A | 362 | N/A |
| 2011 | Peru | Madre de Dios | 0 | 45 | N/A | 35 | N/A | 41 | N/A | 46 | N/A |
| 2011 | Peru | Lima | 0 | 2744 | N/A | 792 | N/A | 482 | N/A | 1639 | N/A |
| 2011 | Peru | Huancavelica | 889 | 224 | -75% | 505 | -43% | 478 | -46% | 318 | -64% |
| 2011 | Peru | Ancash | 803 | 378 | -53% | 588 | -27% | 661 | -18% | 533 | -34% |
| 2011 | Peru | Amazonas | 233 | 158 | -32% | 312 | 34% | 299 | 28% | 211 | -9% |
| 2011 | Peru | Cajamarca | 1369 | 551 | -60% | 1191 | -13% | 1126 | -18% | 763 | -44% |
| 2011 | Peru | Piura | 1280 | 640 | -50% | 624 | -51% | 680 | -47% | 684 | -47% |
| 2011 | Peru | Pasco | 107 | 110 | 3% | 125 | 17% | 140 | 30% | 130 | 21% |
| 2011 | Peru | Callao | 0 | 274 | N/A | 61 | N/A | 19 | N/A | 146 | N/A |
| 2011 | Peru | La Libertad | 638 | 588 | -8% | 622 | -3% | 681 | 7% | 656 | 3% |
| 2011 | Peru | Apurímac | 552 | 176 | -68% | 364 | -34% | 349 | -37% | 242 | -56% |
| 2011 | Peru | Huánuco | 930 | 327 | -65% | 616 | -34% | 591 | -36% | 426 | -54% |
| 2011 | Peru | Tacna | 0 | 99 | N/A | 56 | N/A | 62 | N/A | 86 | N/A |
| 2011 | Peru | Ica | 0 | 243 | N/A | 87 | N/A | 69 | N/A | 160 | N/A |
| 2011 | Peru | Ucayali | 0 | 164 | N/A | 200 | N/A | 222 | N/A | 200 | N/A |
| 2011 | Peru | Puno | 470 | 504 | 7% | 653 | 39% | 742 | 58% | 648 | 38% |
| 2011 | Peru | Loreto | 197 | 405 | 105% | 716 | 264% | 731 | 271% | 550 | 179% |
| 2011 | Peru | Moquegua | 0 | 47 | N/A | 30 | N/A | 34 | N/A | 43 | N/A |
| 2011 | Peru | Lambayeque | 0 | 384 | N/A | 271 | N/A | 323 | N/A | 383 | N/A |
| 2011 | Peru | Ayacucho | 1377 | 266 | -81% | 451 | -67% | 447 | -68% | 340 | -75% |
| 2011 | Peru | Junín | 442 | 486 | 10% | 435 | -2% | 470 | 6% | 496 | 12% |
| 2011 | Peru | Cusco | 667 | 437 | -34% | 647 | -3% | 707 | 6% | 580 | -13% |
| 2011 | Peru | Arequipa | 0 | 355 | N/A | 152 | N/A | 139 | N/A | 258 | N/A |
| 2012 | Peru | Lambayeque | 0 | 809 | N/A | 629 | N/A | 690 | N/A | 785 | N/A |
| 2012 | Peru | Ayacucho | 1478 | 562 | -62% | 923 | -38% | 897 | -39% | 699 | -53% |
| 2012 | Peru | La Libertad | 1496 | 1249 | -16% | 1296 | -13% | 1331 | -11% | 1302 | -13% |
| 2012 | Peru | Tacna | 0 | 211 | N/A | 101 | N/A | 100 | N/A | 161 | N/A |
| 2012 | Peru | Apurímac | 931 | 370 | -60% | 699 | -25% | 694 | -25% | 512 | -45% |
| 2012 | Peru | Huancavelica | 1081 | 473 | -56% | 978 | -10% | 963 | -11% | 685 | -37% |
| 2012 | Peru | Pasco | 334 | 231 | -31% | 273 | -18% | 296 | -11% | 271 | -19% |
| 2012 | Peru | Cajamarca | 3249 | 1138 | -65% | 2437 | -25% | 2454 | -24% | 1733 | -47% |
| 2012 | Peru | Moquegua | 0 | 99 | N/A | 62 | N/A | 67 | N/A | 87 | N/A |
| 2012 | Peru | Amazonas | 1032 | 329 | -68% | 649 | -37% | 648 | -37% | 471 | -54% |
| 2012 | Peru | Junín | 632 | 1032 | 63% | 889 | 41% | 958 | 52% | 1032 | 63% |
| 2012 | Peru | Callao | 0 | 583 | N/A | 125 | N/A | 38 | N/A | 299 | N/A |
| 2012 | Peru | Ica | 0 | 514 | N/A | 144 | N/A | 85 | N/A | 294 | N/A |
| 2012 | Peru | Piura | 2650 | 1348 | -49% | 1493 | -44% | 1589 | -40% | 1501 | -43% |
| 2012 | Peru | Huánuco | 1256 | 685 | -45% | 1238 | -1% | 1218 | -3% | 913 | -27% |
| 2012 | Peru | Arequipa | 0 | 752 | N/A | 412 | N/A | 436 | N/A | 622 | N/A |
| 2012 | Peru | Tumbes | 0 | 150 | N/A | 61 | N/A | 57 | N/A | 106 | N/A |
| 2012 | Peru | Cusco | 1402 | 917 | -35% | 1392 | -1% | 1486 | 6% | 1216 | -13% |
| 2012 | Peru | Ancash | 1282 | 804 | -37% | 1082 | -16% | 1177 | -8% | 1016 | -21% |
| 2012 | Peru | Puno | 727 | 1060 | 46% | 1522 | 109% | 1623 | 123% | 1360 | 87% |
| 2012 | Peru | San Martin | 0 | 593 | N/A | 750 | N/A | 809 | N/A | 717 | N/A |
| 2012 | Peru | Loreto | 3485 | 850 | -76% | 1468 | -58% | 1428 | -59% | 1088 | -69% |
| 2012 | Peru | Ucayali | 0 | 347 | N/A | 333 | N/A | 336 | N/A | 344 | N/A |
| 2012 | Peru | Lima | 0 | 5834 | N/A | 2011 | N/A | 1583 | N/A | 3734 | N/A |
| 2012 | Peru | Madre de Dios | 0 | 95 | N/A | 67 | N/A | 73 | N/A | 88 | N/A |
